# CK2α Overexpression in Colorectal Cancer: Evidence for Sex- and Age-Linked Differences

**DOI:** 10.3390/cancers17172857

**Published:** 2025-08-30

**Authors:** Jana Romy Friedrich, Clara Meier, Guido Plotz, Stefan Zeuzem, Angela Brieger, Sarah J. Overby

**Affiliations:** Biomedical Research Laboratory, Medical Clinic 1, University Hospital, Goethe University Frankfurt, 60596 Frankfurt am Main, Germany; jr.friedrich@med.uni-frankfurt.de (J.R.F.); clmeier@med.uni-frankfurt.de (C.M.); plotz@med.uni-frankfurt.de (G.P.); zeuzem@em.uni-frankfurt.de (S.Z.); overby@med.uni-frankfurt.de (S.J.O.)

**Keywords:** colorectal cancer, casein kinase 2 alpha, CK2, CK2α, female, sex, age

## Abstract

Background/Objectives: Colorectal cancer (CRC) remains a leading cause of cancer-related deaths, with notable sex-specific differences in its incidence, diagnosis, and outcomes. Our previous work identified casein kinase 2 alpha (CK2α) as being capable of impairing DNA mismatch repair (MMR) via phosphorylation of MLH1, thereby increasing the tumor mutational burden. This study aimed to investigate sex-specific differences in CK2α protein expression in CRC. Methods: Immunohistochemical (IHC) analysis was performed on 161 CRC tumors and adjacent normal tissues to quantify the CK2α protein levels. A multi-cohort meta-analysis of proteomic and clinical data was conducted to validate our findings and assess the correlations with age, sex, and relevant signaling pathways. Results: Female CRC patients exhibited significantly higher CK2α expression than male patients, which was confirmed in two independent cohorts. Additionally, CK2α expression was positively correlated with age in female but not male patients. Cross-cohort correlation analyses linked CK2α levels with key proteins involved in estrogen receptor signaling and aging, including DEAD-box helicase 5 (DDX5), histone deacetylase 1 (HDAC1), proliferating cell nuclear antigen (PCNA), prohibitin-2 (PHB2), H/ACA ribonucleoprotein complex subunit 2 (NHP2), and dual-specificity mitogen-activated protein kinase kinase 3 (MAP2K3). Conclusions: CK2α is significantly overexpressed in the tumor tissue of female CRC patients and shows a strong age-related correlation. These findings suggest a sex- and age-specific regulatory mechanism potentially influenced by estrogen signaling or menopause. Such dimorphisms underscore the need for sex-specific strategies in CRC biomarker development and therapy.

## Simple Summary

Colorectal cancer (CRC) affects both men and women, but women are often diagnosed later and may respond differently to treatment. This study focused on the protein casein kinase 2 alpha (CK2α), a key player in multiple cancer-driving pathways. Here, we found that the CK2α levels were higher in CRC tumors from women than from men. We confirmed this phenomenon across two other large patient datasets, and we found that it also correlated with female aging. The increase in CK2α might be linked to changes in hormone levels as women age, such as those occurring during menopause. These findings suggest that women may need different treatment approaches in response to aging and hormone shifts. Understanding these differences could help develop more personalized treatments for CRC patients.

## 1. Introduction

Worldwide, colorectal cancer (CRC) is the third most common cancer (9.6% of all diagnosed cancers in 2022) and the second leading cause of cancer-related deaths (9.3%) [1]. After breast cancer, it is the most diagnosed cancer in European women [2]. Women with CRC tend to be elderly due to lower female endoscopic screening rates [3,4]. Women are also more likely to have right-sided colon cancer lesions, which are associated with more advanced tumor stages [5,6]. However, men have a higher incident rate of CRC than women [7]. Epidemiological and experimental evidence has suggested a protective role of estrogens in CRC, particularly in women, with studies reporting a reduced incidence and improved survival among those exposed to exogenous hormones such as oral contraceptives or hormone replacement therapy [8,9,10]. This contrasts with, e.g., breast and ovarian cancers, where estrogen signaling through estrogen receptor α (ERα) promotes tumorigenesis, as in the colon, estrogen receptor β (ERβ) predominates and has a protective role linked to anti-proliferative and pro-apoptotic effects [11]. Accordingly, higher ERβ expression in CRC tissue has consistently been associated with a favorable prognosis and better survival outcomes [12].

Around 15% of CRCs present high microsatellite instability (MSI-H). This is attributed to dysfunction of the DNA mismatch repair (MMR) system most commonly caused by mutations or hypermethylation of MutL protein homolog 1 (MLH1) [13]. Impairment of MMR proteins is associated with a high mutational burden and is routinely checked for in CRC diagnosis [14]. MMR-deficient and MSI-H CRC patients are often good candidates for immune checkpoint inhibitor therapy (ICI) as this phenotype is a biomarker for chemotherapeutic resistance to drugs like 5-fluorouracil (5-FU), platinum, and methylating agents [14,15,16]. Recently, our group has shown that overexpression of casein kinase 2 alpha (CK2α) increases the levels of phosphorylated MLH1 [17]. Furthermore, we were able to demonstrate that phosphorylated MLH1 impairs MMR activity [18]. This provides evidence for an alternative mechanism by which somatic mutations and tumorigenesis can occur other than the canonical MMR mutational signature.

Casein kinase 2 (CK2) is a serine/threonine kinase with two alpha subunits, α and α’, and two beta subunits, β and β’. CK2 is a regulator of key signal transduction pathways such as Wnt, JAK/STAT, MAPK, and PTEN/PI3K/AKT [19]. Endogenously, the catalytic CK2α subunit is ubiquitously expressed, with elevated levels in highly proliferative tissues including the brain, liver, and gonads [19]. Its abundance is regulated by growth factor signaling, inflammatory mediators, and post-transcriptional mechanisms, such as miRNAs, ensuring control of its activity under physiological conditions [19,20]. Beyond its role in homeostasis, dysregulated CK2α expression has been linked to a broad spectrum of diseases, including neurodegeneration, cardiovascular dysfunction, and chronic inflammation. Because of its involvement in nearly all the hallmarks of cancer [19], dysregulation of CK2 can have carcinogenic downstream consequences [20]. Indeed, CK2 is frequently overexpressed in CRC patient tissue, which correlates with an increased tumor mutational burden and significantly reduced overall survival [17,21]. Aberrant CK2α levels are observed in many cancer types and in hormone-dependent malignancies such as breast, ovarian, and prostate cancers, affecting both female and male patients [19].

In the present work, we investigated a possible sex-specific correlation with CK2α protein expression in CRC by examining a collective of 161 patients. We then compared our results with two previously published independent studies. Finally, we correlated the CK2α expression with sex- and age-specific parameters.

## 2. Materials and Methods

### 2.1. Patients

Formalin-fixed, paraffin-embedded (FFPE) tissue samples of well-characterized colorectal tumors and matched adjacent normal colonic mucosa were obtained from 161 out of 165 patients with CRC from a previously described cohort [17] for use in this study. See Supplementary Figure S1 for a flowchart of the study design. Detailed characteristics of the individual tissue specimens are provided in the original publication. All patients underwent curative-intent colorectal resection. Individuals who had received neoadjuvant chemotherapy were excluded to prevent potential confounding effects of cytoreductive treatment on the tumor genetics. Surgical resections were performed between January 2011 and December 2016 at the University Hospital Frankfurt. The study was approved by the local ethics committee of the University Hospital Frankfurt, and written informed consent was obtained from all participants.

### 2.2. Immunohistochemical Staining

The immunohistochemical (IHC) staining data used in this study were partly obtained from a previously published dataset [17]. For validation and illustrative purposes, additional IHC staining was independently performed to assess the CK2α expression, specifically immunohistochemical staining of the paraffin-embedded colorectal tumor samples, as well as the corresponding adjacent normal mucosa, following standard protocols. In brief, representative FFPE tissue sections (2 µm thick) were cut and mounted on X-tra^®^ microscope slides. To prepare them for the staining procedure, the slides were heated at 70 °C for 10 min. Afterwards, the slides were deparaffinized in 100% xylene, followed by rehydration through a graded ethanol series. Antigen retrieval was performed by heating the sections in a 1 mM ethylenediaminetetraacetic acid (EDTA) buffer (pH 8) (Abcam, ab93680, Cambridge, UK) at 100 °C for 15 min to reverse the formalin-induced protein cross-linking. After cooling, the slides were transferred to phosphate-buffered saline (PBS). Primary antibody incubation followed for 30 min at room temperature (RT) using an anti-CK2α antibody (D-10: sc-365762; Santa Cruz Biotechnology, Dallas, TX, USA) diluted at a ratio of 1:5000 in PBS containing 1% bovine serum albumin (BSA). The sections were then washed in PBS for 2 min—a step repeated after each subsequent incubation. Endogenous peroxidase activity was blocked using 3% hydrogen peroxide (H_2_O_2_) for 5 min at RT. Next, the sections were incubated with a horseradish peroxidase (HRP)-conjugated secondary antibody (K500711-2, Agilent, Santa Clara, CA, USA) for 20 min at RT. Detection was performed using 3,3′-diaminobenzidine (DAB) as a chromogen, applied for 10 min in the dark (K500711-2, Agilent, Santa Clara, CA, USA). Counterstaining was performed using Mayer’s hematoxylin solution (254766.1211, PanReac AppliChem, Darmstadt, Germany). To prepare the slides for foil cover-slipping, an increased ethanol series was applied, ending with 100% xylene. Negative controls were processed in parallel to ensure specificity and rule out nonspecific staining.

### 2.3. Image Processing

Representative images of the immunohistochemical stains were acquired using a digital slide scanner (3DHISTECH, Sysmex, Budapest, Hungary). Image sections of both the tumor tissue and the adjacent normal colorectal mucosa were then created at 10× magnification using Case Viewer software (version 2.4, 3DHISTECH, Sysmex, Budapest, Hungary). Semi-quantitative image analysis was performed using ImageJ software (version 1.53n, NIH). To eliminate nonspecific background signals, a lower threshold of 50 was applied. Mean intensity values were then calculated for each image based on all the pixels exceeding this threshold. Stains were categorized into CK2α-High or CK2α-Low expression groups using the following formulas:

$$Percent\;change=\frac{Tumor\;sample\;-Normal\;sample}{abs\left(Normal\;sample\right)}\times 100$$

Percent change≥20%=CK2α-High;  Percent change<20%=CK2α-Low

### 2.4. Multi-Cohort Meta-Analysis

Colon adenocarcinoma (COAD) clinical and mass spectrometry data was retrieved from the Clinical Proteomic Tumor Analysis Consortium (CPTAC) Pan-Cancer analysis (https://proteomic.datacommons.cancer.gov/pdc/cptac-pancancer, accessed on 14 August 2025) [22,23]. CK2α-High and CK2α-Low categorizations were made using the formula above; however, the threshold was set to 2% to account for the smaller margin of difference found between the normal and tumor tissue in this dataset. COAD and rectum adenocarcinoma (READ) clinical and mass spectrometry data were retrieved from a study by Li et al. [24]. Patients were selected from the validation cohort due to the availability of proteomic data for them and their sex. See Supplementary Figure S1 for a flowchart of the study design.

Estrogen receptor signaling pathway genes were identified using Gene Ontology AmiGO [25,26,27] and STRING protein–protein analysis for estrogen receptor α and estrogen receptor β [28], as well as the following literature: research by Aguilar-Garcia et al. [29] and Fuentes and Silveyra et al. [30]. Menopause-related genes were identified from the following literature: research by Davis et al. [31] and Liu et al. [32]. Aging-related proteins in CRC were identified from research by Diaz-Gay et al. [33], Gong et al. [34], Pretzsch et al. [35], Wang et al. [36], and Yao et al. [37]. A complete list of the genes and their sources can be found in Supplementary Table S1.

### 2.5. Statistical Analysis

To determine the immunohistochemical staining intensities, statistical tests were performed, and bar graphs were generated in Graphpad Prism Software (version 10.1.1 for Mac, GraphPad Software, Boston, MA, USA, www.graphpad.com, accessed on 14 August 2025). Age correlation analysis was performed, and scatter plots were generated in R (version 4.4.2) using the ‘stats’, ‘ggplot2’, and ‘ggpubr’ packages. For the multi-cohort meta-analysis, data from CPTAC PANCAN and Li et al. [24] was imported into and organized in R (version 4.4.2) using ‘dplyr’ and ‘biomaRt’. As well as the mass spectrometry values for the CK2α protein, meta-data was also imported, including the sample IDs, sex, age, and survival rates, when available. All correlation analyses and analyses of survival statistics were performed and all scatter plots and heatmaps were generated using the packages mentioned above as well as ‘Hmisc’, ‘ggcorrplot’, ‘ggsurvfit’, and ‘Survival’. Bar graphs for the CK2α levels and Kaplan–Meier survival curves were generated in Graphpad Prism Software (version 10.1.1).

## 3. Results

### 3.1. Incidence of High CK2α Protein Intensity Is Greater in Female CRC Patients than in Male Patients

**Figure 1:**
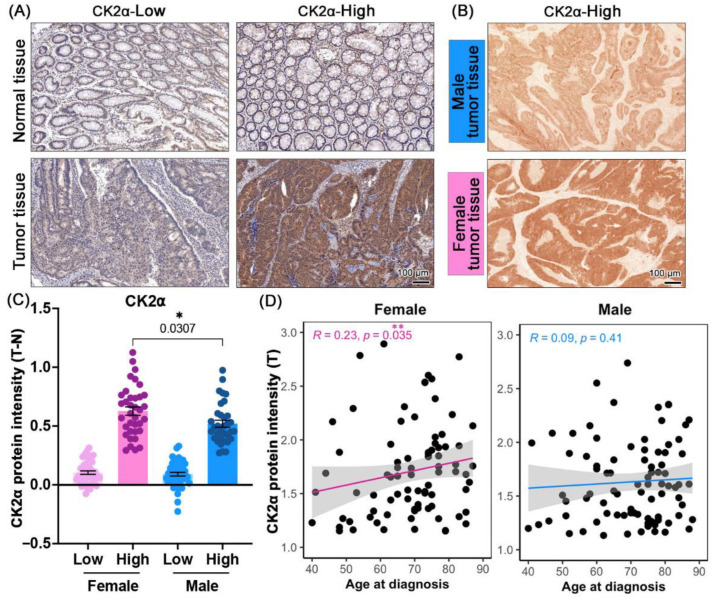
Paraffin-embedded, invasively growing CRC tissue and corresponding surrounding normal tissue analyzed for CK2α expression by immunohistochemical staining. In all panels, dots refer to individual patients. (**A**) Exemplary results are shown representing CK2α-High and CK2α-Low stain phenotypes in tumor tissue along with hematoxylin and eosin (H&E) staining. Matched normal patient tissue is shown for comparison. (**B**) Exemplary images of male vs. female tumor tissue with CK2α-High staining and without H&E staining. (**C**) CK2α protein intensity was quantified from immunohistochemistry stains of patient tumor tissue and normalized to that of normal tissue (T-N). Patient tissue was categorized as CK2α-High if there was 20% increase in intensity compared to normal tissue. Light pink = Female CK2α-Low, Dark pink = Female CK2α-High, Light blue = Male CK2α-Low, Dark blue = Male CK2α-High. (**D**) CK2α protein intensity in tumor tissue (T) correlated with age at diagnosis for female and male patients. Pink and blue lines refer to the respective linear regression performed with the correlation analysis. Error bars represent mean ± SEM. Statistical comparisons were made with Mann–Whitney test. Correlation analysis was performed using Spearman Rho (R) rank correlation. p-values: *p* < 0.05 = *, *p* < 0.01 = **.

Cancerous and adjacent normal tissue from 161 patients was immunologically stained for the CK2α protein. The expression of CK2α has been previously classified into three immunohistochemical phenotypes: (1) High intensity in the nucleus and cytoplasm, (2) high intensity in the nucleus, (3) low intensity in the nucleus and cytoplasm [17]. In order to prepare these results for comparison with studies quantifying CK2α using other groups or methods (i.e., mass spectrometry), a new threshold was set to classify the CK2α intensity as CK2α-High or CK2α-Low. In brief, stains were categorized as CK2α-High when there was a 20% increase or greater in the CK2α intensity in comparison to the matched normal adjacent tissue. The rest were categorized as CK2α-Low. The clinical patient data can be found in Table 1. Representative images of these stains are shown in Figure 1A,B. The intensity was quantified and plotted, comparing the CK2α levels of males and females in the CK2α-High and -Low groups (Figure 1C). Female patients classified as CK2α-High had significantly higher levels of CK2α than men in the same category (*p* = 0.0307).

Finally, we correlated the CK2α protein intensities with the age at diagnosis (Figure 1D). Interestingly, for female patients, we detected a significant correlation (R = 0.23, *p* = 0.035) between higher CK2α expression and a higher age (Figure 1D, left panel). Importantly, this trend was not visible in the male patients (Figure 1D, right panel).

### 3.2. CK2α Protein Levels Are Correlated with Female Aging Across Multiple Clinical Datasets

To verify these results using an external dataset, two individual cohorts were selected for meta-analysis of the CK2α protein levels in CRC patients (Table 1, Supplementary Figure S1). The cohorts were chosen based on the availability of sex- and age-specific proteomics data obtained using mass spectrometry for the CRC patients.

**Figure 2:**
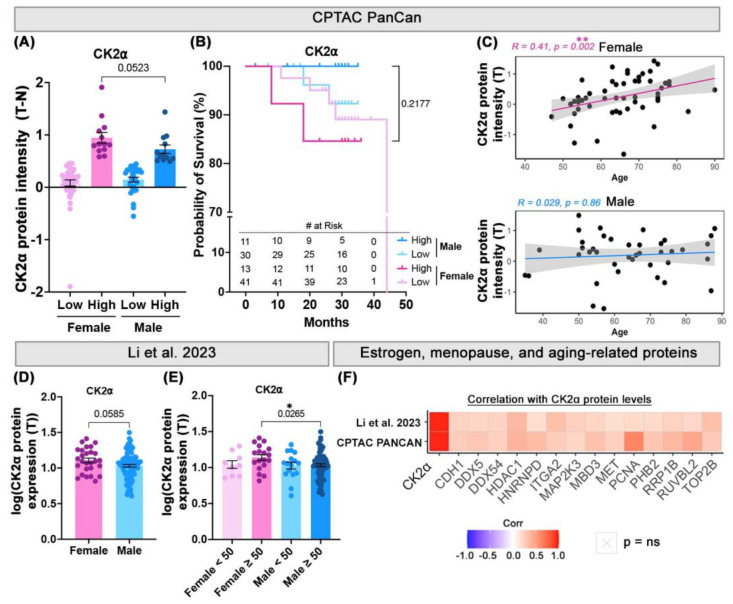
Multi-cohort analysis of sex, survival, and age in relation to CK2α expression in CRC. In all panels, dots refer to individual patients. Clinical and proteomic data was sourced from either Clinical Proteomic Tumor Analysis Consortium (CPTAC) PanCan [22,23] colon cancer patients or colorectal cancer patients from study by Li et al., 2023 [24]. For CPTAC cohort, normalized CK2α protein expression (T-N) was compared between male and females in relation to (**A**) CK2α-High and CK2α-Low intensities and (**B**) survival. Light pink = Female CK2α-Low, Dark pink = Female CK2α-High, Light blue = Male CK2α-Low, Dark blue = Male CK2α-High. Then tumor intensity levels (T) were assessed for correlation with (**C**) age. Pink and blue lines refer to the respective linear regression performed with the correlation analysis. Likewise, CK2α levels of Li et al.’s cohort were compared based on (**D**) sex (Pink = Female, Blue = Male) and (**E**) age of patients. Light pink = Female < 50, Dark pink = Female ≥ 50, Light blue = Male < 50, Dark blue = Male ≥ 50. Protein expression was plotted as log of CK2α value in tumor tissue (T). (**F**) Both cohorts were used to find correlations between CK2α protein levels and estrogen receptor signaling pathway, menopause, and aging-related proteins. Matrix heatmap values are shown for those proteins which were statistically significant and correlated in same direction. Error bars represent mean ± SEM. Panel A shows results of statistical comparisons made with Mann–Whitney test. Panel B shows results of comparison of overall survival in CK2α-High males and CK2α-High females using log-rank Mantel–Cox test. Number of patients at risk is shown on *x*-axis below. Panels C and F show correlations determined using Spearman Rho (R) rank correlation method. To obtain results in panels D and E, data was log-transformed, and statistical comparisons were made with Student’s T-test. *p*-values: *p* < 0.05 = *, *p* < 0.01 = **.

First, the CK2α protein levels were compared in tumor and normal colon tissue from colon cancer patients included in the Clinical Proteomic Tumor Analysis Consortium (CPTAC) Pan-Cancer analysis. Female CK2α-High patients had slightly higher levels than CK2α-High males (*p* = 0.0523) (Figure 2A). CK2α-High females also showed lower overall survival in comparison to both CK2a-High males (*p* = 0.2177) and CK2a-Low males (Figure 2B). Importantly, female patients demonstrated a strong positive correlation (R = 0.41, *p* = 0.002) between their CK2α levels and age that could not be detected in male patients (Figure 2C). These data align with our own IHC data in regard to the female sex specificity of the correlation with age, suggesting a sex-specific role for CK2α, particularly in aging female CRC patients, and a poor prognosis.

The second cohort was sourced from a proteomics study by Li et al. [24] analyzing over 200 CRC patients to discover biomarkers for chemotherapeutic resistance. CK2α-High/Low categorization was impossible for this cohort due to a lack of normal tissue data. The age data was also limited as it was only given in relation to 50 (i.e., <50 or ≥50). Still, of the 124 CRC patients with sex data in this cohort, female patients had slightly higher CK2α protein expression overall (*p* = 0.0585) (Figure 2D). Furthermore, female patients over 50 years old also showed significantly higher CK2α levels than male patients in the same age group (*p* = 0.0265) (Figure 2E). Again, these results corroborate the pattern seen in our own data, as well as the CPTAC data, regarding the sex- and age-linked overexpression of CK2α.

Due to the strong correlations between CK2α and aging in female patients, we hypothesized that CK2α may play a role in or be influenced by estrogen receptor signaling, menopause, and aging. Importantly, proteomic data was not available for ERα or ERβ in either of the cohorts. Therefore, a list of genes involved in these pathways was collected and cross-referenced with the corresponding protein levels across both cohorts (Supplementary Table S1). Significant correlations are shown in a correlation matrix heatmap in Figure 2F, while the correlations of all the proteins can be found in Supplementary Figure S2 and Supplementary Table S2. Of note, DEAD-box helicase 5 (DDX5) and 54 (DDX54), histone deacetylase 1 (HDAC1), heterogeneous nuclear ribonucleoprotein D0 (HNRNPD), integrin alpha-2 (ITGA2), methyl-CpG-binding domain protein 3 (MBD3), proliferating cell nuclear antigen (PCNA), prohibitin-2 (PHB2), and RuvB-like 2 (RUVBL2) were significantly correlated with CK2α expression across all the cohorts and are all part of the estrogen signaling pathway or the cellular response to estradiol. Likewise, aging-related proteins significantly correlated with CK2α across all the datasets included cadherin-1 (CDH1), hepatocyte growth factor receptor (MET), dual-specificity mitogen-activated protein kinase kinase 3 (MAP2K3), ribosomal RNA processing protein 1 homolog B (RRP1B), and DNA topoisomerase 2-beta (TOP2B). These correlations point to the currently unexplored interaction between CK2α and female hormones, menopausal status, and age.

## 4. Discussion

The detection of MMR deficiency in CRC plays a critical role in informing chemotherapy decisions [15]. Indeed, patients with metastatic CRC and MSI-H due to defective MMR are eligible for ICI treatment due to their high mutational burden [14]. Our group has shown previously that MMR can also be effectively blocked through phosphorylation of MLH1 by CK2α [18]. CK2α overexpression increases this phosphorylation and contributes to non-canonical MMR deactivation and increased tumor mutation rates [17]. In the present study, we demonstrated that women showed significantly higher levels of CK2α in their tumor tissue compared to men, which was also the trend in two independent datasets. The analysis of the current cohort and the meta-analysis also revealed a positive correlation between CK2α and female age, which prompted an analysis of estrogen receptor signaling, menopause, and aging-related factors. Of note, the estrogen-related proteins DDX5/54, HDAC1, HNRNPD, ITGA2, MBD3, PCNA, PHB2, and RUVBL2 were all significantly correlated with CK2α expression across all the cohorts. The same pattern was seen in the aging-related proteins CDH1, MET, MAP2K3, RRP1B, and TOP2B. Interestingly, many of these proteins have been described as being altered in the context of cancer and as being associated with a poor prognosis.

DDX5, for example, is upregulated in many cancers, including CRC, and plays a master role in transcription factor activation and RNA metabolism [38]. Specifically, DDX5 and its paralog DDX17 are key players in the estrogen and androgen signaling pathways [39]. DDX5 is also a known splicing regulator of other estrogen co-activators such as mediator subunit 1 (MED1), as well as corepressor nuclear corepressor 2 (N-CoR2), also known as the silencing mediator of retinoic acid and thyroid hormone receptor (SMRT) protein. In the same family of RNA helicases, DDX54 also interacts with estrogen receptors. Furthermore, it is also upregulated in CRC and contributes to tumor proliferation via the p65/AKT pathway [40].

HDAC1, in turn, is a recognized transcriptional suppressor of estrogen receptor α (ERα) [41] and is associated with breast cancer [42]. Indeed, histone deacetylases have even been tested for use as therapeutic targets in breast cancer cells through HDAC inhibition (HDACi) [43]. This type of therapy has also shown success in CRC cells, which also show HDAC1 upregulation [44]. ITGA2 also plays a role in breast cancer by promoting metastasis [45,46].

PCNA is a known marker for cell proliferation and plays a role in DNA replication and repair as well as cell cycle regulation [47]. In one study comparing pre- and post-menopausal breast cancer patients, PCNA showed higher expression in the post-menopausal group and a significant correlation with HER-2/neu expression in the same group [48]. ERα upregulates PCNA and enhances breast cancer cell proliferation [49]. Interestingly, PCNA is associated with a poor prognosis in CRC [50] and plays a key role in recruiting MMR proteins for subsequent repair [51].

PHB2, complexed with PHB1, is a protein found in the mitochondrial membrane; however, it has also been found in other areas of the cell such as the cytoplasm and nucleus [52]. PHB2 plays a role in cell survival and is implicated in various cancers and diseases. In breast cancer, PHB2 represses estrogen and acts as a tumor suppressor [53]. Notably, PHB2 is able to bind specifically to the ERα wildtype (WT) and common ERα mutants found in breast cancer [54]. Interestingly, PHB2 is also known to interact with HDAC1 and recruit it to the nucleus [53].

In regard to aging-related proteins, MET codes for c-Met protein and is a known proto-oncogene involved in many cancers [55]. MET overexpression is associated with poor survival in non-small-cell lung cancer [56], while c-Met has been tested for use as a therapeutic target using c-Met inhibitors in gastric cancer [57]. C-Met is also known as a marker for cellular senescence [58] and was one of the most upregulated proteins in elderly CRC patients compared to young patients in two separate proteomics studies [34,36].

MAP2K3, also known as MKK3, is associated with cellular senescence [59] and has been implicated in Alzheimer’s disease and age-related memory decline [60]. It is also associated with tumor progression and invasiveness in many cancers, including colorectal cancer [61,62,63]. In tumor cells mutant for p53, an upregulation of MAP2K3 is associated with tumor proliferation and survival, and MAP2K3 has been identified as a potential therapeutic target [64].

Finally, NHP2 is a component of the telomerase complex. Mutations in this gene are linked to the premature aging syndrome dyskeratosis congenita [65]. NHP2 is overexpressed in elderly patients and is associated with a poor prognosis in CRC [34,66]. The protein was found to be associated with high levels of CK2α in the CPTAC cohort and the study by Li et al. [24] (Supplementary Figure S2).

Greater overall survival in women with colon cancer has been associated with the pre-menopause stage, suggesting a potential protective role of hormones at this age [7]. Post-menopause, female levels of the estrogen hormone tend to fall to similar levels to those of men [7]. Nevertheless, the literature remains controversial, as several large prospective studies have failed to confirm consistent associations of estradiol levels with the CRC incidence [67,68]. These inconsistencies highlight the complexity of estrogen signaling in CRC and suggest that its effect may vary among molecular subgroups [10] and that other pathways may modulate its effects [69]. This also raises the question of whether the older age at CRC diagnosis observed in women is attributable to lower rates of early endoscopy, biological effects related to post-menopausal hormonal changes, or both. In this context, our findings on CK2α are of particular interest, as CK2α interacts with multiple signaling networks linked to hormone receptor pathways and could therefore influence how estrogen signaling translates into tumor-suppressive or tumor-promoting outcomes in CRC.

Between 2000 and 2022, only seven studies were published analyzing sex-specific differences in the context of CRC [70]. In a study of Food and Drug Administration (FDA)-approved drug trials, women were largely underrepresented across many types of cancer, including colorectal cancer [71]. In an analysis of over 30,000 patients, 5-FU showed significantly higher toxicity to women in colon cancer [72]. But even with this knowledge, women with colon and rectal cancer are treated less aggressively and have less pre-operative radiotherapy than men [3,73]. These statistics and the results of the present study emphasize the importance of investigating sexual dimorphisms in precision medicine, particularly when it comes to biomarker identification, hormonal levels, menopause status, drug dosages, and toxicity.

## 5. Conclusions

This study highlights a significant sex- and age-specific overexpression of CK2α in female CRC patients. The findings suggest a potential link between the CK2α levels and female aging, underscoring the importance of incorporating sex-specific biomarkers in CRC research and treatment strategies. Further investigation into CK2α’s role could improve personalized therapies for women with CRC.

## Figures and Tables

**Table 1:** Clinical features of the three analyzed CRC cohorts.

	Ulreich et al. 2022 [17]			CPTAC PanCan			Li et al. 2023 [24]
	Total	Low	High	Total	Low	High	Total
	(n = 161; 100.00%)	(*n* = 91; 56.52%)	(*n* = 70; 43.48%)	(*n* = 95; 100.00%)	(*n* = 71; 74.74%)	(*n* = 24; 25.26%)	(*n* = 124; 100.00%)
**Sex category**							
Female	*n* = 81 (50.31%)	*n* = 45 (49.45%)	*n* = 36 (51.43%)	*n* = 54 (56.84%)	*n* = 41 (57.75%)	*n* = 13 (54.17%)	*n* = 27 (21.77%)
Male	*n* = 80 (49.69%)	*n* = 46 (50.55%)	*n* = 34 (48.57%)	*n* = 41 (43.16%)	*n* = 30 (42.25%)	*n* = 11 (45.83%)	*n* = 97 (78.23%)
**Median age at diagnosis (IQR)**	72 (18)	70 (19.5)	74.5 (15.75)	65 (18)	67 (18)	59 (13)	
**Localization**							
Distal	*n* = 59 (36.65%)	*n* = 33 (36.26%)	*n* = 26 (37.14%)				
Proximal	*n* = 95 (59.00%)	*n* = 55 (60.44%)	*n* = 40 (57.14%)				
Left side of colon							*n* = 32 (25.81%)
Right side of colon							*n* = 12 (9.68%)
Rectum							*n* = 76 (61.29%)
Unknown/other	*n* = 7 (4.35%)	*n* = 3 (3.30%)	*n* = 4 (5.72%)				*n* = 4 (3.23%)
**Year of diagnosis and operation**							
2008	*n* = 1 (0.62%)	*n* = 0 (0.00%)	*n* = 1 (1.43%)				
2010	*n* = 1 (0.62%)	*n* = 1 (1.10%)	*n* = 0 (0.00%)				
2011	*n* = 9 (5.59%)	*n* = 5 (5.49%)	*n* = 4 (5.72%)				
2012	*n* = 31 (19.25%)	*n* = 19 (20.88%)	*n* = 12 (17.14%)				
2013	*n* = 36 (22.36%)	*n* = 22 (24.18%)	*n* = 14 (20.00%)				
2014	*n* = 32 (19.88%)	*n* = 18 (19.78%)	*n* = 14 (20.00%)				
2015	*n* = 23 (14.29%)	*n* = 12 (13.19%)	*n* = 11 (15.71%)				
2016	*n* = 28 (17.39%)	*n* = 14 (15.38%)	*n* = 14 (20.00%)				
**Tumor**							
pT1/pT1a	*n* = 12 (7.45%)	*n* = 9 (9.89%)	*n* = 3 (4.29%)	*n* = 0 (0.00%)	*n* = 0 (0.00%)	*n* = 0 (0.00%)	
pT2	*n* = 31 (19.26%)	*n* = 15 (16.48%)	*n* = 16 (22.86%)	*n* = 13 (13.68%)	*n* = 9 (12.68%)	*n* = 4 (16.67%)	
pT3	*n* = 93 (57.76%)	*n* = 54 (59.34%)	*n* = 39 (55.71%)	*n* = 71 (74.74%)	*n* = 55 (77.46%)	*n* = 16 (66.67%)	
pT4/pT4a/pT4b	*n* = 25 (15.53%)	*n* = 13 (14.29%)	*n* = 12 (17.14%)	*n* = 11 (11.58%)	*n* = 7 (9.86%)	*n* = 4 (16.67%)	
**Metastases**							
M0	*n* = 121 (75.16%)	*n* = 69 (75.82%)	*n* = 52 (74.29%)	*n* = 45 (47.37%)	*n* = 35 (49.30%)	*n* = 10 (41.67%)	*n* = 39 (31.45%)
M1	*n* = 40 (24.84%)	*n* = 22 (24.18%)	*n* = 18 (25.71%)	*n* = 7 (7.37%)	*n* = 5 (7.04%)	*n* = 2 (8.33%)	*n* = 83 (66.94%)
MX				*n* = 43 (45.26%)	*n* = 31 (43.66%)	*n* = 12 (50.00%)	*n* = 2 (1.61%)
**UICC Stage**							
I	*n* = 37 (22.98%)	*n* = 20 (21.98%)	*n* = 17 (24.29%)	*n* = 10 (10.53%)	*n* = 7 (9.86%)	*n* = 3 (12.50%)	
II/IIA/IIB/IIC	*n* = 47 (29.19%)	*n* = 32 (35.16%)	*n* = 15 (21.43%)	*n* = 39 (41.05%)	*n* = 29 (40.85%)	*n* = 10 (41.67%)	
III/IIIA/IIIB/IIIC	*n* = 39 (24.23%)	*n* = 20 (21.98%)	*n* = 19 (27.14%)	*n* = 39 (41.05%)	*n* = 30 (42.25%)	*n* = 9 (37.50%)	
IV/IVA/IVB	*n* = 38 (23.60%)	*n* = 19 (20.88%)	*n* = 19 (27.14%)	*n* = 7 (74.74%)	*n* = 5 (7.04%)	*n* = 2 (8.33%)	
**Histology**							
Adenocarcinoma	*n* = 148 (91.93%)	*n* = 80 (87.91%)	*n* = 68 (97.14%)	*n* = 76 (80.00%)	*n* = 55 (77.46%)	*n* = 21 (87.50%)	
Mucinous adenocarcinoma	*n* = 10 (6.21%)	*n* = 8 (8.79%)	*n* = 2 (2.86%)	*n* = 18 (18.95%)	*n* = 16 (22.54%)	*n* = 2 (8.33%)	
Mucin-producing adenocarcinoma	*n* = 2 (1.24%)	*n* = 2 (2.20%)	*n* = 0 (0.00%)				
Neuroendocrine carcinoma	*n* = 1 (0.62%)	*n* = 1 (1.10%)	*n* = 0 (0.00%)				
Not reported				*n* = 1 (1.05%)	*n* = 0 (0.00%)	*n* = 1 (4.17%)	
**Race**							
American Indian				*n* = 1 (1.05%)	*n* = 1 (1.41%)	*n* = 0 (0.00%)	
Asian				*n* = 16 (16.84%)	*n* = 11 (15.49%)	*n* = 5 (20.83%)	
Black or African American				*n* = 7 (74.74%)	*n* = 5 (7.04%)	*n* = 2 (8.33%)	
White/Hispanic				*n* = 3 (3.16%)	*n* = 2 (2.82%)	*n* = 1 (4.17%)	
White/not Hispanic				*n* = 65 (68.42%)	*n* = 49 (69.01%)	*n* = 16 (16.67%)	
Unknown				*n* = 2 (2.11%)	*n* = 2 (2.82%)	*n* = 0 (0.00%)	

## Data Availability

The results shown here are based in whole or in part upon data generated by the National Cancer Institute Clinical Proteomic Tumor Analysis Consortium (CPTAC, https://pdc.cancer.gov, accessed on 14 August 2025) (Study: PDC000116) and original research by Li et al. [24], https://doi.org/10.1016/j.xcrm.2023.101311 (accessed on 14 August 2025). The raw data supporting the conclusions of this article will be made available by the authors on request.

## Supplementary Materials

The following supporting information can be downloaded at https://www.mdpi.com/article/10.3390/cancers17172857/s1: Supplementary Figure S1: Flowchart of the study design; Supplementary Figure S2: Correlation heatmap for all proteins; Supplementary Table S1: Genes of interest; Supplementary Table S2: Correlation statistics.

## Abbreviations

The following abbreviations are used in this manuscript:

5-FU	5-fluorouracil
BSA	Bovine serum albumin
CDH1	Cadherin-1
CK2	Casein kinase 2
CK2α	Casein kinase 2 alpha
COAD	Colon adenocarcinoma
CPTAC	Clinical Proteomic Tumor Analysis Consortium
CRC	Colorectal cancer
DAB	3,3′-diaminobenzidine
DDX5	DEAD-box helicase 5
DDX54	DEAD-box helicase 54
EDTA	Ethylenediaminetetraacetic acid
ERα	Estrogen receptor α (protein)
ERβ	Estrogen receptor β (protein)
ESR1	Estrogen receptor α (gene)
ESR2	Estrogen receptor β (gene)
FDA	Food and Drug Administration
FFPE	Formalin-fixed, paraffin-embedded
H&E	Hematoxylin and eosin
H_2_O_2_	Hydrogen peroxide
HDAC1	Histone deacetylase 1
HDACi	HDAC inhibition
HNRNPD	Heterogeneous nuclear ribonucleoprotein D0
HRP	Horseradish peroxidase
ICI	Immune checkpoint inhibitor
IHC	Immunohistochemistry/immunohistochemical
ITGA2	Integrin alpha-2
MAP2K3	Dual-specificity mitogen-activated protein kinase kinase 3
MBD3	Methyl-CpG-binding domain protein 3
MED1	Mediator subunit 1
MET	Hepatocyte growth factor receptor
MLH1	MutL protein homolog 1
MMR	DNA mismatch repair
MSI-H	Microsatellite instability
N-CoR2	Corepressor nuclear corepressor 2
NHP2	H/ACA ribonucleoprotein complex subunit 2
PBS	Phosphate-buffered saline
PCNA	Proliferating cell nuclear antigen
PHB2	Prohibitin-2
READ	Rectum adenocarcinoma
RRP1B	Ribosomal RNA processing protein 1 homolog B
RT	Room temperature
RUVBL2	RuvB-like 2
SMRT	Retinoid/thyroid hormone receptors
TOP2B	DNA topoisomerase 2-beta
WT	Wildtype
