# Supplementary material for: CK2α Overexpression in Colorectal Cancer: Evidence for Sex- and Age-Linked Differences
Source: Cancers (Basel). 2025 Aug 30;17(17):2857. doi: 10.3390/cancers17172857 (PMC12427210; doi:10.3390/cancers17172857)
Supplement: Supplementary file 1 [file cancers-17-02857-s001.zip › Table S1 Genes of interest.pdf]

Table S1: Genes of interest

**Proteins**	**Association**	**Source**
ABCB1	response to estradiol, cellular response to estradiol stimulus	GO:0032355, GO:0071392
ABCC9	response to estrogen	GO:0043627
ABL1	cellular senescence	GO:0090398
ADAMTS1	ovulation cycle	GO:0042698
ADCYAP1R1	response to estradiol	GO:0032355
ADH1B	Age-associated proteins in CRC	https://doi.org/10.1016/j.jprot.2023.104863
ADNP	estrous cycle, ovulation cycle	GO:0044849, GO:0042698
AFP	ovulation cycle	GO:0042698
AHR	ESR1/ESR2 STRING	https://string-db.org/cgi/network?taskId=b2JjW4eS8vzu&sessionId=boSOH3HBDGPl
AIFM1	response to estradiol, cellular response to estradiol stimulus	GO:0032355, GO:0071392
AKR1B15	estrogen metabolic process	GO:0008210
ALDH1A2	response to estradiol	GO:0032355
AMH	Menopause, ovulation cycle	https://doi.org/10.1016/j.cell.2023.08.016, GO:0042698
APC	Age and geography associated proteins in CRC, Age-associated proteins in CRC	https://doi.org/10.1038/s41586-025-09025-8, https://doi.org/10.1016/j.jprot.2023.104863
APP	Menopause - TF activated 40-49 age group	https://doi.org/10.3389/fendo.2023.1004245
AR	estrogen receptor signaling pathway, regulation of intracellular estrogen receptor signaling pathway, estrogen response element binding, response to estrogen, cellular response to estrogen stimulus	GO:0030520, GO:0033146, GO:0034056, GO:0043627, GO:0071391
AREG	response to estradiol	GO:0032355

ARID5A	nuclear estrogen receptor binding, response to estrogen, cellular response to estrogen stimulus	GO:0030331, GO:0043627, GO:0071391
ARID5B	Menopause - Follicular cell differentiation, Maintain granulosa	https://doi.org/10.3389/fendo.2023.1004245
ARNT2	response to estradiol	GO:0032355
ARPC1B	response to estradiol, response to estrogen	GO:0032355, GO:0043627
ARRDC1	Age-associated proteins in CRC	https://doi.org/10.1016/j.jprot.2023.104863
ARSB	response to estrogen	GO:0043627
ASH2L	response to estrogen	GO:0043627
ASS1	response to estradiol	GO:0032355
ATM	Age-associated proteins in CRC, cellular senescence	https://doi.org/10.1016/j.mcpro.2021.100115, GO:0090398
ATP5PO	estradiol binding	GO:1903924
AXL	ovulation cycle	GO:0042698
B3GNT3	BC + CRC overlap	https://pubmed.ncbi.nlm.nih.gov/39671411/
BCAS3	response to estrogen, cellular response to estrogen stimulus	GO:0043627, GO:0071391
BGLAP	response to estrogen	GO:0043627
BMAL1	cellular senescence	GO:0090398
BMP7	response to estradiol	GO:0032355
BMPR1B	ovulation cycle	GO:0042698
BRCA1	regulation of intracellular estrogen receptor signaling pathway	GO:0033146
BRCA2	cellular senescence	GO:0090398
CA12	estrous cycle, ovulation cycle	GO:0044849, GO:0042698
CALR	response to estradiol, cellular senescence	GO:0032355, GO:0090398
CARM1	regulation of intracellular estrogen receptor signaling pathway	GO:0033146

CASC3	Age-associated proteins in CRC	https://doi.org/10.1016/j.jprot.2023.104863
CASP2	ovulation cycle	GO:0042698
CASP3	ovulation cycle, response to estradiol	GO:0042698, GO:0032355
CASP8	Age-associated proteins in CRC, response to estradiol	https://doi.org/10.1016/j.mcpro.2021.100115, GO:0032355
CASP9	response to estradiol	GO:0032355
CAT	response to estradiol	GO:0032355
CAV1	response to estrogen	GO:0043627
CCDC62	estrogen receptor signaling pathway, nuclear estrogen receptor binding, ESR1/ESR2 STRING, response to estradiol, cellular response to estradiol stimulus	GO:0030520, GO:0030331, GO:0032355, GO:0071392
CCNA2	response to estradiol, cellular response to estradiol stimulus	GO:0032355, GO:0071392
CCND1	response to estradiol, response to estrogen	GO:0032355, GO:0043627
CD24	response to estrogen	GO:0043627
CD38	Age-associated proteins in CRC, response to estradiol	https://doi.org/10.1016/j.mcpro.2021.100115, GO:0032355
CD4	response to estradiol	GO:0032355
CDH1	Age and metastasis associated proteins in CRC	https://doi.org/10.1016/j.canep.2022.102112
CDK1	Age-associated proteins in CRC	https://doi.org/10.1016/j.mcpro.2021.100115, https://doi.org/10.1016/j.jprot.2023.104863
CDK12	regulation of intracellular estrogen receptor signaling pathway, response to estrogen, cellular response to estrogen stimulus	GO:0033146, GO:0043627, GO:0071391
CDK2	cellular senescence	GO:0090398
CDK5RAP1	Age-associated proteins in CRC	https://doi.org/10.1016/j.mcpro.2021.100115
CDKN1A	cellular senescence	GO:0090398
CDKN1B	cellular senescence	GO:0090398

CDKN2A	cellular senescence	GO:0090398
CDKN2B	cellular senescence	GO:0090398
CEACAM5	Age-associated proteins in CRC	https://doi.org/10.1016/j.jprot.2023.104863
CEACAM6	Age-associated proteins in CRC	https://doi.org/10.1016/j.jprot.2023.104863
CFLAR	response to estradiol, cellular response to estradiol stimulus	GO:0032355, GO:0071392
CGA	ovulation cycle	GO:0042698
CHD1	Age-associated proteins in CRC	https://doi.org/10.1016/j.jprot.2023.104863
CHST10	estrogen metabolic process	GO:0008210
CITED1	response to estrogen	GO:0043627
CITED2	response to estrogen, cellular senescence	GO:0043627, GO:0090398
CITED4	response to estrogen	GO:0043627
CLDN18	response to estrogen, cellular response to estrogen stimulus	GO:0043627, GO:0071391
CNOT1	nuclear estrogen receptor binding, regulation of intracellular estrogen receptor signaling pathway	GO:0030331, GO:0033146
CNOT2	regulation of intracellular estrogen receptor signaling pathway	GO:0033146
CNOT9	regulation of intracellular estrogen receptor signaling pathway	GO:0033146
COL1A1	response to estradiol	GO:0032355
COMP	cellular senescence	GO:0090398
CRH	response to estrogen	GO:0043627
CRHBP	response to estradiol, cellular response to estradiol stimulus, response to estrogen, cellular response to estrogen stimulus	GO:0032355, GO:0071392, GO:0043627, GO:0071391
CRYAB	response to estradiol	GO:0032355
CSN1S1	response to estradiol	GO:0032355
CTNNA1	response to estrogen	GO:0043627

CTNNB1	nuclear estrogen receptor binding, response to estradiol	GO:0030331, GO:0032355
CXCL3	Age and metastasis associated proteins in CRC	https://doi.org/10.1016/j.canep.2022.102112
CYP11A1	estrogen receptor signaling pathway	https://doi.org/10.1016/bs.apcsb.2019.01.001
CYP17A1	estrogen receptor signaling pathway	https://doi.org/10.1016/bs.apcsb.2019.01.001
CYP19A1	estrogen receptor signaling pathway, response to estradiol, regulation of estradiol secretion, estrogen metabolic process	https://doi.org/10.1016/bs.apcsb.2019.01.001, GO:0032355, GO:2000864, GO:0008210
CYP1A1	estrogen metabolism, estrogen metabolic process	https://doi.org/10.1016/bs.apcsb.2019.01.001, GO:0008210
CYP1A2	estrogen metabolism, estrogen metabolic process	https://doi.org/10.1016/bs.apcsb.2019.01.001, GO:0008210
CYP1B1	estrogen metabolism, estrogen metabolic process	https://doi.org/10.1016/bs.apcsb.2019.01.001, GO:0008210
CYP27B1	response to estrogen	GO:0043627
CYP2C8	estrogen metabolic process	GO:0008210
CYP2C9	estrogen metabolic process	GO:0008210
CYP2D6	estrogen metabolic process	GO:0008210
CYP3A4	estrogen metabolic process	GO:0008210
CYP3A5	estrogen metabolic process	GO:0008210
CYP3A7	estrogen metabolic process	GO:0008210
CYP7B1	estrogen receptor signaling pathway, regulation of intracellular estrogen receptor signaling pathway	GO:0030520, GO:0033146
DCAF1	nuclear estrogen receptor binding	GO:0030331
DCAF13	nuclear estrogen receptor binding	GO:0030331
DCN	Menopause - Maintain granulosa, Age-associated proteins in CRC	https://doi.org/10.3389/fendo.2023.1004245, https://doi.org/10.1016/j.mcpro.2021.100115
DDRGK1	regulation of intracellular estrogen receptor signaling pathway	GO:0033146
DDX17	estrogen receptor signaling pathway	GO:0030520

DDX18	response to estradiol, cellular response to estradiol stimulus	GO:0032355, GO:0071392
DDX5	estrogen receptor signaling pathway	GO:0030520
DDX54	estrogen receptor signaling pathway, nuclear estrogen receptor binding	GO:0030520, GO:0030331
DEFA1	estrogen receptor signaling pathway, Age-associated proteins in CRC	GO:0030520, https://doi.org/10.1016/j.mcpro.2021.100115
DEFA3	estrogen receptor signaling pathway, Age-associated proteins in CRC	GO:0030520, https://doi.org/10.1016/j.mcpro.2021.100115
DHH	response to estradiol, response to estrogen	GO:0032355, GO:0043627
DHRS11	estrogen metabolic process	GO:0008210
DLX2	Age biomarkers in CRC	https://doi.org/10.1038/s41598-021-01850-x
DNAAF4	nuclear estrogen receptor binding, regulation of intracellular estrogen receptor signaling pathway	GO:0030331, GO:0033146
DNAJA3	cellular senescence	GO:0090398
DNMT3A	response to estradiol	GO:0032355
DRD2	response to estradiol	GO:0032355
DUSP1	response to estradiol	GO:0032355
ECRG4	cellular senescence	GO:0090398
EEF2	response to estradiol	GO:0032355
EGFR	response to estradiol, cellular response to estradiol stimulus, Age and metastasis associated proteins in CRC	GO:0032355, GO:0071392, https://doi.org/10.1016/j.canep.2022.102112
EGLN2	estrogen receptor signaling pathway	GO:0030520
EGR1	Menopause - TF activated 40-49 age group, estrous cycle, ovulation cycle	https://doi.org/10.3389/fendo.2023.1004245, GO:0044849, GO:0042698
EIF1	Menopause - Maintain granulosa	https://doi.org/10.3389/fendo.2023.1004245
EIF4A1	Age-associated proteins in CRC	https://doi.org/10.1016/j.mcpro.2021.100115
ENDOG	response to estradiol	GO:0032355

ENO2	response to estradiol	GO:0032355
EP300	response to estrogen	GO:0043627
EPO	response to estrogen	GO:0043627
EREG	ovulation cycle	GO:0042698
ERRFI1	response to estradiol	GO:0032355
ESR1	estrogen receptor signaling pathway, nuclear estrogen receptor binding, ESR1/ESR2 STRING, response to estradiol, cellular response to estradiol stimulus, cellular response to estrogen stimulus, estrogen response element binding, response to estrogen, ovulation cycle	GO:0030520, GO:0030331, GO:0032355, GO:0071392, GO:0071391, GO:0034056, GO:0043627, GO:0042698
ESR2	estrogen receptor signaling pathway, ESR1/ESR2 STRING, response to estradiol, cellular response to estradiol stimulus, estrogen response element binding	GO:0030520, GO:0032355, GO:0071392, GO:0034056
ESRRA	estrogen response element binding	GO:0034056
ESRRB	estrogen response element binding	GO:0034056
ESRRG	estrogen response element binding	GO:0034056
EWSR1	Age-associated proteins in CRC	https://doi.org/10.1016/j.mcpro.2021.100115
EZH2	response to estradiol	GO:0032355
F7	response to estradiol, response to estrogen	GO:0032355, GO:0043627
FAM210B	response to estradiol, cellular response to estradiol stimulus	GO:0032355, GO:0071392
FBXO4	cellular senescence	GO:0090398
FGF10	response to estradiol	GO:0032355
FLT3	Age and metastasis associated proteins in CRC	https://doi.org/10.1016/j.canep.2022.102112
FOXA1	regulation of intracellular estrogen receptor signaling pathway, response to estradiol	GO:0033146, GO:0032355
FOXL2	nuclear estrogen receptor binding	GO:0030331

FOXN4	Menopause - TF activated 30-39 age group	https://doi.org/10.3389/fendo.2023.1004245
FOXO3	ovulation cycle	GO:0042698
FOXR1	Menopause - TF activated 30-39 age group	https://doi.org/10.3389/fendo.2023.1004245
FSHB	Menopause, ovulation cycle	https://doi.org/10.1016/j.cell.2023.08.016, GO:0042698
FSHR	Menopause, regulation of intracellular estrogen receptor signaling pathway, ovulation cycle	https://doi.org/10.1016/j.cell.2023.08.016, GO:0033146, GO:0042698
FZD4	ovulation cycle	GO:0042698
GABRB1	ovulation cycle	GO:0042698
GAL	response to estrogen	GO:0043627
GAS2	ovulation cycle	GO:0042698
GATA3	response to estrogen	GO:0043627
GATA6	response to estrogen	GO:0043627
GATM	Age-associated proteins in CRC	https://doi.org/10.1016/j.jprot.2023.104863
GBA1	response to estrogen	GO:0043627
GDF10	ovulation cycle	GO:0042698
GH1	response to estradiol	GO:0032355
GHR	response to estradiol	GO:0032355
GHRHR	response to estrogen	GO:0043627
GHRL	response to estrogen	GO:0043627
GHSR	response to estradiol	GO:0032355
GINS3	cellular senescence	GO:0090398
GLI1	Menopause - TF activated 40-49 age group	https://doi.org/10.3389/fendo.2023.1004245
GNRH1	Menopause, estrous cycle, ovulation cycle	https://doi.org/10.1016/j.cell.2023.08.016, GO:0044849, GO:0042698

GNRHR	Menopause	https://doi.org/10.1016/j.cell.2023.08.016
GPER1	estrogen receptor signaling pathway, ESR1/ESR2 STRING, response to estradiol, cellular response to estradiol stimulus	https://doi.org/10.1016/bs.apcsb.2019.01.001, GO:0032355, GO:0071392
GPI	response to estradiol	GO:0032355
GPR149	ovulation cycle	GO:0042698
GPR30	estrogen receptor signaling pathway	https://doi.org/10.1016/bs.apcsb.2019.01.001
GPX1	response to estradiol	GO:0032355
GPX4	response to estradiol	GO:0032355
GREM1	BC + CRC overlap	https://pubmed.ncbi.nlm.nih.gov/39671411/
GRIA1	response to estradiol	GO:0032355
GSTA1	Age-associated proteins in CRC, Menopause - Follicular cell differentiation	https://doi.org/10.1016/j.mcpro.2021.100115, https://doi.org/10.3389/fendo.2023.1004245
GSTM3	response to estrogen	GO:0043627
GSTP1	response to estradiol	GO:0032355
GTF2H4	Age-associated proteins in CRC	https://doi.org/10.1016/j.jprot.2023.104863
H2AZ1	response to estradiol, cellular response to estradiol stimulus	GO:0032355, GO:0071392
HAS2	estrous cycle, ovulation cycle	GO:0044849, GO:0042698
HDAC1	regulation of intracellular estrogen receptor signaling pathway	GO:0033146
HDAC2	regulation of intracellular estrogen receptor signaling pathway	GO:0033146
HDAC6	regulation of intracellular estrogen receptor signaling pathway	GO:0033146
HMGA1	Age-associated proteins in CRC, cellular senescence	https://doi.org/10.1016/j.mcpro.2021.100115, GO:0090398
HMGA2	cellular senescence	GO:0090398
HMGB1	Menopause - Follicular cell differentiation	https://doi.org/10.3389/fendo.2023.1004245

HNF1A	Menopause - TF activated 30-39 age group	https://doi.org/10.3389/fendo.2023.1004245
HNRNPD	response to estradiol, cellular response to estradiol stimulus	GO:0032355, GO:0071392
HOXA10	response to estrogen	GO:0043627
HOXA11	response to estrogen	GO:0043627
HPGD	response to estradiol	GO:0032355
HPSE2	Age biomarkers in CRC	https://doi.org/10.1038/s41598-021-01850-x
HRAS	cellular senescence	GO:0090398
HSD17B1	estrogen receptor signaling pathway, estradiol binding, estrogen metabolic process	https://doi.org/10.1016/bs.apcsb.2019.01.001, GO:1903924, GO:0008210
HSD17B10	Age-associated proteins in CRC, estrogen metabolic process	https://doi.org/10.1016/j.mcpro.2021.100115, GO:0008210
HSD17B11	estrogen metabolic process	GO:0008210
HSD17B12	estrogen metabolic process	GO:0008210
HSD17B2	estrogen metabolic process	GO:0008210
HSD17B4	estrogen metabolic process	GO:0008210
HSD17B7	estrogen metabolic process	GO:0008210
HSD17B8	estrogen metabolic process	GO:0008210
HSD3B1	estrogen metabolic process	GO:0008210
HSD3B2	estrogen receptor signaling pathway	https://doi.org/10.1016/bs.apcsb.2019.01.001
HSF1	response to estradiol, cellular response to estradiol stimulus	GO:0032355, GO:0071392
HSP90AA1	response to estrogen	GO:0043627
HSP90AB1	Menopause - Maintain granulosa	https://doi.org/10.3389/fendo.2023.1004245
HTR5A	response to estradiol	GO:0032355

ID2	cellular senescence	GO:0090398
IFITM1	Age and metastasis associated proteins in CRC	https://doi.org/10.1016/j.canep.2022.102112
IGFBP2	response to estradiol, response to estrogen	GO:0032355, GO:0043627
IHH	response to estradiol	GO:0032355
IL10	response to estradiol, cellular response to estradiol stimulus	GO:0032355, GO:0071392
ING1	Age-associated proteins in CRC	https://doi.org/10.1016/j.jprot.2023.104863
INHBA	ovulation cycle	GO:0042698
INHBB	Menopause	https://doi.org/10.1016/j.cell.2023.08.016
ISL1	nuclear estrogen receptor binding, regulation of intracellular estrogen receptor signaling pathway	GO:0030331, GO:0033146
ITGA2	response to estradiol, cellular response to estradiol stimulus	GO:0032355, GO:0071392
ITGAM	response to estradiol	GO:0032355
JUN	Menopause - Follicular cell differentiation	https://doi.org/10.3389/fendo.2023.1004245
KANK2	Age-associated proteins in CRC, regulation of intracellular estrogen receptor signaling pathway	https://doi.org/10.1016/j.mcpro.2021.100115, GO:0033146
KAT5	response to estradiol, cellular response to estradiol stimulus, cellular senescence	GO:0032355, GO:0071392, GO:0090398
KAT6A	cellular senescence	GO:0090398
KDM5B	regulation of estradiol secretion	GO:2000864
KIF18A	response to estradiol, cellular response to estradiol stimulus	GO:0032355, GO:0071392
KMT2D	regulation of intracellular estrogen receptor signaling pathway, response to estrogen	GO:0033146, GO:0043627
KRT18	Menopause - Follicular cell differentiation	https://doi.org/10.3389/fendo.2023.1004245
KRT19	response to estrogen	GO:0043627
LATS1	nuclear estrogen receptor binding, regulation of intracellular estrogen receptor signaling pathway	GO:0030331, GO:0033146

LBH	regulation of intracellular estrogen receptor signaling pathway	GO:0033146
LCOR	nuclear estrogen receptor binding, response to estradiol, cellular response to estradiol stimulus	GO:0030331, GO:0032355, GO:0071392
LEF1	nuclear estrogen receptor binding	GO:0030331
LEP	ovulation cycle, response to estradiol	GO:0042698, GO:0032355
LHB	Menopause	https://doi.org/10.1016/j.cell.2023.08.016
LHCGR	Menopause, ovulation cycle	https://doi.org/10.1016/j.cell.2023.08.016, GO:0042698
LILRB2	Age-associated proteins in CRC	https://doi.org/10.1016/j.jprot.2023.104863
LMNA	cellular senescence	GO:0090398
MAGEA2	cellular senescence	GO:0090398
MAP1B	response to estradiol	GO:0032355
MAP2K1	cellular senescence	GO:0090398
MAP2K3	cellular senescence	GO:0090398
MAP2K4	cellular senescence	GO:0090398
MAP2K6	cellular senescence	GO:0090398
MAP2K7	cellular senescence	GO:0090398
MAP3K5	cellular senescence	GO:0090398
MAPK10	cellular senescence	GO:0090398
MAPK11	cellular senescence	GO:0090398
MAPK14	cellular senescence	GO:0090398
MAPK8	cellular senescence	GO:0090398
MAPK9	cellular senescence	GO:0090398
MAPKAPK5	cellular senescence	GO:0090398

MBD2	response to estradiol	GO:0032355
MBD3	response to estradiol	GO:0032355
MBD4	response to estradiol	GO:0032355
MDK	estrous cycle, ovulation cycle	GO:0044849, GO:0042698
MDM2	cellular response to estrogen stimulus, response to estrogen	GO:0071391, GO:0043627
MED1	nuclear estrogen receptor binding, regulation of intracellular estrogen receptor signaling pathway	GO:0030331, GO:0033146
MET	Age-associated proteins in CRC	https://doi.org/10.1016/j.mcpro.2021.100115, https://doi.org/10.1016/j.jprot.2023.104863
METTL7B	Age-associated proteins in CRC	https://doi.org/10.1016/j.mcpro.2021.100115
MGME1	Age-associated proteins in CRC	https://doi.org/10.1016/j.jprot.2023.104863
MIF	cellular senescence	GO:0090398
MME	response to estrogen	GO:0043627
MMP14	response to estrogen	GO:0043627
MMP15	response to estradiol	GO:0032355
MMP19	ovulation cycle	GO:0042698
MMP2	response to estradiol, cellular response to estradiol stimulus, response to estrogen, ovulation cycle	GO:0032355, GO:0071392, GO:0043627, GO:0042698
MMS19	nuclear estrogen receptor binding	GO:0030331
MNT	cellular senescence	GO:0090398
MORF4L2	Age-associated proteins in CRC	https://doi.org/10.1016/j.jprot.2023.104863
MSTN	ovulation cycle, response to estrogen	GO:0042698, GO:0043627
MSX2	response to estradiol, cellular response to estradiol stimulus	GO:0032355, GO:0071392
MTCO2	Menopause - Follicular cell differentiation	https://doi.org/10.3389/fendo.2023.1004245

MYBL2	Menopause - TF activated 30-39 age group	https://doi.org/10.3389/fendo.2023.1004245
MYH11	Age-associated proteins in CRC	https://doi.org/10.1016/j.jprot.2023.104863
MYL9	Age-associated proteins in CRC	https://doi.org/10.1016/j.jprot.2023.104863
MYOD1	response to estradiol, cellular response to estradiol stimulus	GO:0032355, GO:0071392
MYOG	response to estradiol, cellular response to estradiol stimulus	GO:0032355, GO:0071392
NCOA1	estrogen receptor signaling pathway, nuclear estrogen receptor binding, response to estradiol, estrous cycle, ovulation cycle	GO:0030520, GO:0030331, GO:0032355, GO:0044849, GO:0042698
NCOA3	response to estradiol, cellular response to estradiol stimulus	GO:0032355, GO:0071392
NCOA4	cellular response to estrogen stimulus, estrogen receptor signaling pathway, response to estrogen	GO:0071391, GO:0030520, GO:0043627
NCOA6	nuclear estrogen receptor binding	GO:0030331
NCOA7	ESR1/ESR2 STRING	https://string-db.org/cgi/network?taskId=b2JjW4eS8vzu&sessionId=boSOH3HBDGPl
NCOR2	response to estradiol, estrous cycle, ovulation cycle	GO:0032355, GO:0044849, GO:0042698
NDUFS6	cellular senescence	GO:0090398
NFYC	Age-associated proteins in CRC	https://doi.org/10.1016/j.mcpro.2021.100115
NHLH2	ovulation cycle	GO:0042698
NHP2	Age-associated proteins in CRC	https://doi.org/10.1016/j.mcpro.2021.100115
NKX3-1	nuclear estrogen receptor binding	GO:0030331
NOC2L	Age-associated proteins in CRC	https://doi.org/10.1016/j.mcpro.2021.100115
NOL7	Age-associated proteins in CRC	https://doi.org/10.1016/j.mcpro.2021.100115, https://doi.org/10.1016/j.jprot.2023.104863
NOL8	Age-associated proteins in CRC	https://doi.org/10.1016/j.jprot.2023.104863
NOP2	Age-associated proteins in CRC	https://doi.org/10.1016/j.mcpro.2021.100115

NOS3	ovulation cycle	GO:0042698
NOTCH1	ovulation cycle	GO:0042698
NOTCH4	ovulation cycle	GO:0042698
NPM1	cellular senescence	GO:0090398
NPPC	ovulation cycle	GO:0042698
NPR2	ovulation cycle	GO:0042698
NPY5R	ovulation cycle	GO:0042698
NR2F2	response to estradiol	GO:0032355
NR3C1	estrogen response element binding	GO:0034056
NR3C2	estrogen response element binding	GO:0034056
NR5A1	ovulation cycle	GO:0042698
NR5A2	ovulation cycle	GO:0042698
NR6A1	estrogen response element binding	GO:0034056
NRIP1	nuclear estrogen receptor binding, response to estradiol, cellular response to estradiol stimulus, ovulation cycle	GO:0030331, GO:0032355, GO:0071392, GO:0042698
NSD1	nuclear estrogen receptor binding	GO:0030331
NSMCE2	cellular senescence	GO:0090398
NUP62	cellular senescence	GO:0090398
OCSTAMP	cellular response to estrogen stimulus, response to estrogen	GO:0071391, GO:0043627
OLFM4	BC + CRC overlap	https://pubmed.ncbi.nlm.nih.gov/39671411/
OPA1	cellular senescence	GO:0090398
OPRK1	estrous cycle, response to estrogen, ovulation cycle	GO:0044849, GO:0043627, GO:0042698
OTX2	Menopause - TF activated 30-39 age group	https://doi.org/10.3389/fendo.2023.1004245

OXT	response to estradiol	GO:0032355
PADI2	estrogen receptor signaling pathway, nuclear estrogen receptor binding	GO:0030520, GO:0030331
PAGR1	nuclear estrogen receptor binding, regulation of intracellular estrogen receptor signaling pathway	GO:0030331, GO:0033146
PAK1	regulation of intracellular estrogen receptor signaling pathway	GO:0033146
PARP1	nuclear estrogen receptor binding, regulation of intracellular estrogen receptor signaling pathway	GO:0030331, GO:0033146
PCNA	nuclear estrogen receptor binding, response to estradiol, estrous cycle, ovulation cycle	GO:0030331, GO:0032355, GO:0044849, GO:0042698
PCOLCE2	Age biomarkers in CRC	https://doi.org/10.1038/s41598-021-01850-x
PDE3A	estrogen binding	GO:0099130
PDE4B	BC + CRC overlap	https://pubmed.ncbi.nlm.nih.gov/39671411/
PDGFRA	estrogen metabolic process, ovulation cycle	GO:0008210, GO:0042698
PELP1	cellular response to estrogen stimulus, response to estrogen	GO:0071391, GO:0043627
PENK	response to estradiol	GO:0032355
PGR	estrogen response element binding, ovulation cycle	GO:0034056, GO:0042698
PHB2	estrogen receptor signaling pathway, nuclear estrogen receptor binding, regulation of intracellular estrogen receptor signaling pathway	GO:0030520, GO:0030331, GO:0033146
PHF8	Age-associated proteins in CRC	https://doi.org/10.1016/j.jprot.2023.104863
PIN1	Age-associated proteins in CRC	https://doi.org/10.1016/j.mcpro.2021.100115
PLA2R1	cellular senescence	GO:0090398
PLEKHA1	estrogen metabolic process, ovulation cycle	GO:0008210, GO:0042698
PML	cellular senescence	GO:0090398
POSTN	response to estradiol	GO:0032355
POU4F1	response to estradiol, cellular response to estradiol stimulus	GO:0032355, GO:0071392

POU4F2	estrogen receptor signaling pathway, response to estradiol, cellular response to estradiol stimulus	GO:0030520, GO:0032355, GO:0071392
PPAN	Age-associated proteins in CRC	https://doi.org/10.1016/j.mcpro.2021.100115
PPARGC1B	estrogen receptor signaling pathway, nuclear estrogen receptor binding	GO:0030520, GO:0030331
PPID	nuclear estrogen receptor binding	GO:0030331
PPP1R9B	response to estradiol, cellular response to estradiol stimulus	GO:0032355, GO:0071392
PRELP	cellular senescence	GO:0090398
PRKAA1	response to estrogen	GO:0043627
PRKCA	response to estradiol	GO:0032355
PRKCD	cellular senescence	GO:0090398
PRMT2	nuclear estrogen receptor binding	GO:0030331
PRMT6	cellular senescence	GO:0090398
PSEN1	Age-associated proteins in CRC	https://doi.org/10.1016/j.mcpro.2021.100115
PTCH1	response to estradiol	GO:0032355
PTGFR	response to estradiol	GO:0032355
PTN	estrous cycle, ovulation cycle	GO:0044849, GO:0042698
PTPRN	ovulation cycle	GO:0042698
PTX3	ovulation cycle	GO:0042698
RAMP3	response to estradiol, cellular response to estradiol stimulus	GO:0032355, GO:0071392
RARA	cellular response to estrogen stimulus, response to estradiol, response to estrogen	GO:0071391, GO:0032355, GO:0043627
RBBP5	response to estrogen	GO:0043627
RBFOX2	estrogen receptor signaling pathway	GO:0030520
RBP1	Menopause - Granulosa differentiation	https://doi.org/10.3389/fendo.2023.1004245

RCAN1	response to estrogen	GO:0043627
RDH8	estrogen metabolic process	GO:0008210
RERG	nuclear estrogen receptor binding	GO:0030331
RGS9	response to estradiol	GO:0032355
ROBO2	ovulation cycle	GO:0042698
RRP1B	Age-associated proteins in CRC	https://doi.org/10.1016/j.mcpro.2021.100115
RUVBL2	response to estradiol, cellular response to estradiol stimulus	GO:0032355, GO:0071392
SAA1	Age and metastasis associated proteins in CRC	https://doi.org/10.1016/j.canep.2022.102112
SAFB	estrogen receptor signaling pathway	GO:0030520
SCAPER	ovulation cycle	GO:0042698
SERPINB5	Age-associated proteins in CRC	https://doi.org/10.1016/j.jprot.2023.104863
SERPINB9	cellular response to estrogen stimulus, response to estrogen	GO:0071391, GO:0043627
SERPINE2	Menopause - Granulosa differentiation	https://doi.org/10.3389/fendo.2023.1004245
SERPINF1	ovulation cycle	GO:0042698
SFR1	cellular response to estrogen stimulus, response to estrogen	GO:0071391, GO:0043627
SFRP1	response to estradiol, cellular response to estradiol stimulus, response to estrogen, cellular response to estrogen stimulus	GO:0032355, GO:0071392, GO:0043627, GO:0071391
SGPL1	estrogen metabolic process, ovulation cycle	GO:0008210, GO:0042698
SIRT1	ovulation cycle, cellular senescence	GO:0042698, GO:0090398
SKP2	regulation of intracellular estrogen receptor signaling pathway	GO:0033146
SLC10A1	response to estrogen	GO:0043627
SLC26A6	ovulation cycle	GO:0042698
SLC26A6	estrous cycle	GO:0044849

SLC34A1	response to estradiol	GO:0032355
SLC34A2	response to estrogen	GO:0043627
SLC6A4	response to estradiol	GO:0032355
SLIT2	ovulation cycle	GO:0042698
SLIT3	ovulation cycle	GO:0042698
SMAD1	Menopause - TF activated 40-49 age group	https://doi.org/10.3389/fendo.2023.1004245
SMAD6	response to estrogen	GO:0043627
SMAD7	Menopause - TF activated 40-49 age group	https://doi.org/10.3389/fendo.2023.1004245
SMC5	cellular senescence	GO:0090398
SMC6	cellular senescence	GO:0090398
SOCS2	response to estradiol	GO:0032355
SP1	cellular response to estrogen stimulus, response to estrogen	GO:0071391, GO:0043627
SPI1	cellular senescence	GO:0090398
SPP1	regulation of estradiol secretion	GO:2000864
SRARP	nuclear estrogen receptor binding, regulation of intracellular estrogen receptor signaling pathway	GO:0030331, GO:0033146
SRC	regulation of intracellular estrogen receptor signaling pathway	GO:0033146
SRD5A1	response to estradiol, cellular response to estradiol stimulus, response to estrogen	GO:0032355, GO:0071392, GO:0043627
SRF	cellular senescence	GO:0090398
SSTR1	response to estradiol, cellular response to estradiol stimulus	GO:0032355, GO:0071392
SSTR2	response to estradiol, cellular response to estradiol stimulus	GO:0032355, GO:0071392
STAT3	response to estradiol	GO:0032355
STAT5A	ovulation cycle	GO:0042698

STAT5B	ovulation cycle, response to estradiol	GO:0042698, GO:0032355
STRN	nuclear estrogen receptor binding	GO:0030331
STRN3	regulation of intracellular estrogen receptor signaling pathway, response to estradiol	GO:0033146, GO:0032355
STXBP1	response to estradiol	GO:0032355
SULT1A1	estrogen metabolic process	GO:0008210
SULT1E1	estrogen metabolic process	GO:0008210
TACC1	nuclear estrogen receptor binding	GO:0030331
TACC3	Age-associated proteins in CRC	https://doi.org/10.1016/j.jprot.2023.104863
TACR1	response to estradiol	GO:0032355
TACR3	response to estradiol	GO:0032355
TADA3	estrogen receptor signaling pathway	GO:0030520
TAF10	nuclear estrogen receptor binding	GO:0030331
TAF7	estrogen receptor signaling pathway	GO:0030520
TAF8	Age-associated proteins in CRC	https://doi.org/10.1016/j.jprot.2023.104863
TBX2	cellular senescence	GO:0090398
TBX3	cellular senescence	GO:0090398
TERF2	cellular senescence	GO:0090398
TGFB1	response to estradiol	GO:0032355
TGFB2	ovulation cycle	GO:0042698
TGFB3	ovulation cycle	GO:0042698
TIPARP	estrogen metabolic process	GO:0008210
TMSB10	Menopause - Granulosa differentiation	https://doi.org/10.3389/fendo.2023.1004245

TMSB4X	Menopause - Granulosa differentiation	https://doi.org/10.3389/fendo.2023.1004245
TNFAIP6	ovulation cycle	GO:0042698
TOE1	Age-associated proteins in CRC	https://doi.org/10.1016/j.mcpro.2021.100115
TOP2B	cellular senescence	GO:0090398
TP53	cellular senescence	GO:0090398
TP63	regulation of intracellular estrogen receptor signaling pathway, cellular senescence	GO:0033146, GO:0090398
TRIM24	cellular response to estrogen stimulus, estrogen response element binding, response to estrogen	GO:0071391, GO:0034056, GO:0043627
TRIM25	response to estrogen	GO:0043627
TRIP4	estrogen receptor signaling pathway, nuclear estrogen receptor binding	GO:0030520, GO:0030331
TSPO	Age-associated proteins in CRC	https://doi.org/10.1016/j.mcpro.2021.100115
TXNIP	response to estradiol	GO:0032355
TYRO3	ovulation cycle	GO:0042698
UBA5	regulation of intracellular estrogen receptor signaling pathway	GO:0033146
UBR5	estrogen receptor signaling pathway	GO:0030520
UCN	response to estradiol	GO:0032355
UFL1	regulation of intracellular estrogen receptor signaling pathway	GO:0033146
UFM1	regulation of intracellular estrogen receptor signaling pathway	GO:0033146
UFSP2	regulation of intracellular estrogen receptor signaling pathway	GO:0033146
UGT1A1	estrogen metabolic process	GO:0008210
UGT1A3	estrogen metabolic process	GO:0008210
UGT1A7	estrogen metabolic process	GO:0008210
UGT2B10	estrogen metabolic process	GO:0008210

UGT2B11	estrogen metabolic process	GO:0008210
UGT2B15	estrogen metabolic process	GO:0008210
UGT2B17	estrogen metabolic process	GO:0008210
UGT2B28	estrogen metabolic process	GO:0008210
UGT2B4	estrogen metabolic process	GO:0008210
UGT2B7	estrogen metabolic process	GO:0008210
UHRF1	Age-associated proteins in CRC	https://doi.org/10.1016/j.mcpro.2021.100115
ULK3	cellular senescence	GO:0090398
VCP	Age-associated proteins in CRC	https://doi.org/10.1016/j.mcpro.2021.100115
VPS11	regulation of intracellular estrogen receptor signaling pathway	GO:0033146
VPS18	regulation of intracellular estrogen receptor signaling pathway	GO:0033146
WBP2	cellular response to estrogen stimulus, nuclear estrogen receptor binding, regulation of intracellular estrogen receptor signaling pathway, response to estrogen	GO:0071391, GO:0030331, GO:0033146, GO:0043627
WIPI1	nuclear estrogen receptor binding	GO:0030331
WNT16	cellular senescence	GO:0090398
WNT7A	response to estradiol, response to estrogen	GO:0032355, GO:0043627
WNT8B	response to estradiol	GO:0032355
WRN	cellular senescence	GO:0090398
XBP1	nuclear estrogen receptor binding	GO:0030331
ZDHHC21	ESR1/ESR2 STRING	https://string-db.org/cgi/network?taskId=b2JjW4eS8vzu&sessionId=boSOH3HBDGPl
ZMIZ1	cellular senescence	GO:0090398
ZNF366	nuclear estrogen receptor binding, regulation of intracellular estrogen	GO:0030331, GO:0033146, GO:0043627

	receptor signaling pathway, response to estrogen	
ZNF703	response to estradiol, cellular response to estradiol stimulus	GO:0032355, GO:0071392
ZNF830	ovulation cycle	GO:0042698
