# Supplementary material for: CK2α Overexpression in Colorectal Cancer: Evidence for Sex- and Age-Linked Differences
Source: Cancers (Basel). 2025 Aug 30;17(17):2857. doi: 10.3390/cancers17172857 (PMC12427210; doi:10.3390/cancers17172857)
Supplement: Supplementary file 1 [file cancers-17-02857-s001.zip › Table S2 Correlation statistics.pdf]

Table S2: Correlation statistics

	CPTAC PANCAN		Li *et al.* 2023	
**Protein**	**Rho**	**P-Value**	**Rho**	**P-Value**
CSNK2A1	1	0	1	0
ABL1	-0,23198906	0,085350727	0,001273378	0,988801153
ADH1B	0,053336951	0,605773986	-0,22486231	0,012044312
APC	-0,25965117	0,049033569	NA	NA
ARRDC1	-0,20360516	0,125285005	0,025933238	0,774951861
ATM	-0,32246337	0,001354656	-0,01539575	0,865235385
BRCA2	-0,42857143	0,289403225	-0,11298887	0,211499202
CASC3	-0,10065725	0,377427777	-0,05010753	0,580489248
CDH1	0,274172545	0,006868622	0,242555468	0,006641875
CDK1	0,189609333	0,064274505	-0,04017624	0,657742845
CDK2	-0,08499729	0,410290717	0,138523997	0,124950298
CDK5RAP1	0,060417843	0,789402335	-0,04856346	0,592221789
CDKN1B	-0,30950694	0,004171717	0,007384862	0,935121781
CDKN2A	0,062337662	0,788364073	0,033850511	0,708975209
CEACAM5	-0,0027401	0,978861945	0,146731707	0,103906743
CEACAM6	0,02800469	0,799173024	0,21875059	0,014651854
CHD1	0,149172545	0,146899608	-0,0278368	0,75892133
COMP	-0,28894466	0,00430166	NA	NA
DNAJA3	0,09700217	0,347118676	0,125570417	0,164638682
EIF4A1	0,33685567	0,000791049	0,156626279	0,082353446
EWSR1	0,134441129	0,191576085	0,347915028	7,51E-05
FBXO4	-0,13953488	0,366337836	0,103112045	0,254445061
FLT3	0,207172911	0,047533272	0,041018682	0,651031853
GATM	-0,15465274	0,132452903	0,107317585	0,2354783
GINS3	0,1	0,64199323	0,159355678	0,077085444
GTF2H4	0,133675214	0,515032388	0,070552025	0,436186862
HMGA1	0,028974596	0,779299596	-0,09912195	0,273386758
HMGA2	0,178136619	0,098790195	0,022856468	0,801061101
HRAS	-0,34701697	0,07041754	-0,05649725	0,533118146
IFITM1	-0,04072165	0,693635706	0,18179701	0,043303038
ING1	-0,03652174	0,865463193	0,053793265	0,552927093
KAT6A	-0,11640482	0,340838617	0,049066651	0,588386532
LILRB2	-0,10407192	0,424764368	0,084386871	0,351419755
LMNA	-0,24385513	0,016654511	-0,16259323	0,071192015
MAP2K1	0,092213782	0,371548126	-0,03897404	0,667366987
MAP2K3	0,215504612	0,034971294	0,248503541	0,005386077
MAP2K4	0,122172145	0,235699283	-0,03325256	0,713890482
MAP2K6	-0,09937602	0,335389688	0,035616106	0,694532114
MAP2K7	-0,10243788	0,365892653	0,063901141	0,480755807
MAP3K5	-0,17495411	0,156758196	0,039308869	0,664680991
MAPK14	0,008918913	0,931272364	0,222426436	0,01303039
MAPK8	-0,10535403	0,334341271	0,04094514	0,651616592

MAPK9	-0,00992946	0,923507554	0,14272856	0,113793706
MAPKAPK5	0,384942043	0,000231864	NA	NA
MET	0,253621812	0,012654949	0,179971676	0,045484514
MGME1	0,395830485	0,002530248	NA	NA
MIF	-0,13044717	0,205228956	0,227411487	0,011082931
MORF4L2	-0,0205236	0,842672555	0,065549327	0,469488004
MYH11	-0,25328269	0,012778349	0,121321794	0,179510468
MYL9	-0,34722039	0,000528304	0,088503541	0,328335406
NDUFS6	-0,02832339	0,784137599	0,274781814	0,00201149
NFYC	0,237429501	0,021929134	0,028808812	0,750774484
NHP2	0,243326099	0,01689931	0,147143149	0,102930009
NOC2L	0,245550733	0,01589043	-0,13530219	0,134055011
NOL7	0,223982637	0,028251401	0,154181409	0,087313494
NOL8	0,252084757	0,060892402	0,030100559	0,739990299
NOP2	0,182921867	0,074445303	0,035354839	0,696662619
NPM1	0,189283776	0,064742023	0,101859953	0,260289276
NSMCE2	-0,17034129	0,274792134	NA	NA
NUP62	-0,19401178	0,058217424	0,271729347	0,002266704
OPA1	0,008803581	0,932158964	-0,09231786	0,307834499
PHF8	0,142477775	0,213362158	-0,15119638	0,093688996
PIN1	-0,35623983	0,000367594	0,341331235	0,000104633
PML	-0,01395822	0,89262997	0,102105429	0,259136332
PRELP	-0,25058414	0,013798194	-0,15721794	0,081187675
PRKCD	-0,20318774	0,047087774	-0,0752919	0,405914652
PSEN1	-0,02830315	0,814751793	0,16297117	0,070528487
RRP1B	0,367756375	0,000227704	0,237515448	0,00790275
SAA1	0,100176343	0,33149263	-0,05998275	0,508117869
SERPINB5	0,013388497	0,896987868	0,059600315	0,510830972
SMC5	0,198380567	0,232493672	0,258008659	0,003814405
SMC6	-0,1016956	0,324174421	0,101711345	0,260988951
TACC3	0,195564516	0,291729783	-0,11315775	0,210812929
TAF8	-0,08421053	0,724103644	0,171596787	0,056698396
TERF2	-0,0318797	0,78455789	0,035072701	0,698965965
TOE1	0,048762138	0,688518718	-0,08473882	0,349407379
TOP2B	0,276776994	0,006335888	0,330309992	0,000179213
TP53	-0,03445378	0,844229108	0,149641666	0,097152701
TSPO	-0,00249593	0,980745157	0,001359581	0,988043089
UHRF1	0,184820944	0,071433623	0,171707317	0,056536811
ULK3	-0,31295547	0,052396902	0,105587001	0,243159894
VCP	0,277482366	0,006198052	0,078457907	0,386403416
WRN	0,164835165	0,590478989	0,082614006	0,361666492
ZMIZ1	0,074178246	0,505113189	0,045155982	0,618485934
ABCB1	-0,02076764	0,845085265	0,111540519	0,217450417
ABCC9	-0,16869565	0,430702262	NA	NA
ADNP	0,17457949	0,088904213	-0,17131078	0,057118273
AHR	-0,14952094	0,223612982	0,111184744	0,218930301

AIFM1	0,193868692	0,058406679	0,080849725	0,372045236
ALDH1A2	-0,03609023	0,879931196	0,061129819	0,500025299
ARPC1B	0,025515464	0,805090281	-0,14555468	0,106741112
ARSB	-0,3653554	0,000251988	-0,03274907	0,718038327
ASH2L	0,017010309	0,869341523	-0,13745397	0,127919769
ASS1	-0,00614487	0,95261832	-0,05992132	0,508553165
ATP5PO	0,209698861	0,040309316	NA	NA
AXL	-0,5	0,666666667	0,001286516	0,988685616
B3GNT3	0,225910895	0,02945279	0,049284028	0,586733221
BCAS3	-0,07702131	0,535584677	-0,09414859	0,298298665
CA12	-0,11356484	0,270593701	-0,12160504	0,178489329
CALR	-0,13707271	0,182947226	0,20702439	0,021053094
CARM1	0,199755833	0,051024347	0,271471282	0,00228957
CASP2	0,221105683	0,034169598	0,143240554	0,112489829
CASP3	-0,12395551	0,228880261	0,075768138	0,402943131
CASP8	0,167335563	0,112872441	0,23407081	0,008882101
CASP9	0,006896552	0,971676459	0,151774629	0,092425982
CAT	-0,19177971	0,061227356	0,012619984	0,889361266
CAV1	-0,15888497	0,122057403	0,017554681	0,846555039
CCNA2	0,016611296	0,91684258	-0,03451111	0,703558893
CD38	-0,37642558	0,000156886	0,10590716	0,241725837
CD4	-0,14348971	0,24306428	-0,11212743	0,215024656
CDK12	0,020930062	0,843020859	0,064890872	0,473972138
CLDN18	0,01978022	0,946489227	-0,15548681	0,084636337
CNOT1	0,258993489	0,010834237	-0,05350747	0,555041393
CNOT2	0,054204223	0,611866051	0,222358533	0,01305886
CNOT9	-0,13369552	0,194073634	NA	NA
COL1A1	-0,36747151	0,000230467	0,030640441	0,735497859
CRYAB	-0,1744981	0,08905552	0,108059795	0,23223636
CTNNA1	0,370496473	0,000202639	-0,10551062	0,243502881
CTNNB1	0,28559414	0,004793663	0,151773407	0,092428639
CYP1B1	0,5	0,666666667	NA	NA
CYP3A5	0,182694873	0,186076877	-0,15488277	0,085866849
CYP7B1	-0,15718628	0,285988909	0,054251652	0,549544086
DCAF1	-0,05394077	0,601693668	NA	NA
DCAF13	0,198453925	0,055180036	0,136550758	0,130468254
DDRGK1	0,228526858	0,02512411	-0,01896459	0,834400768
DDX17	0,162520898	0,113637874	0,422741149	1,00E-06
DDX18	0,254367878	0,012387119	0,165450826	0,066298358
DDX5	0,29114216	0,004003943	0,218851298	0,014605192
DDX54	0,253664704	0,013626681	0,227704333	0,010976921
DEFA3	0,115233706	0,263569524	0,133268569	0,140057559
DHRS11	-0,04540152	0,660486466	0,02855434	0,752904708
EEF2	0,437561042	8,28E-06	0,030885917	0,733458156
EGFR	0,056660336	0,583469324	0,118797797	0,188799944
ENDOG	-0,04426207	0,668500443	0,049810841	0,582735274

ENO2	-0,10252306	0,320232502	0,209265146	0,019670928
EP300	0,013279978	0,897818295	0,081677548	0,367152886
ESRRA	0,18470552	0,219122687	0,153997191	0,087696668
EZH2	-0,25294118	0,344563031	0,100480523	0,266833303
FAM210B	-0,10541126	0,443716516	-0,04408186	0,626868066
FOXL2	-0,05623127	0,664216913	NA	NA
FOXO3	-0,30837438	0,10362155	0,032337534	0,721434798
GAL	-0,52205882	0,031584827	-0,08306817	0,359024074
GATA6	-0,07719298	0,753443345	-0,08278188	0,360688374
GBA1	0,193157193	0,059355215	NA	NA
GPI	0,301749864	0,002810148	0,040321007	0,656587649
GPX1	-0,21129951	0,038773831	0,09147443	0,312294043
GPX4	-0,01543679	0,881335376	0,178335169	0,047517356
GREM1	-0,24567282	0,015836606	0,218005287	0,015001236
GSTM3	-0,2076777	0,042320352	-0,15471282	0,086215614
GSTP1	0,00849159	0,934557783	0,282429583	0,00148237
H2AZ1	-0,07542755	0,46514791	NA	NA
HDAC1	0,39454149	6,96E-05	0,372443745	2,05E-05
HDAC2	0,379896907	0,000134747	-0,05075059	0,575634929
HDAC6	-0,11677971	0,257173865	-0,04129032	0,648873843
HNRNPD	0,343014107	0,000623389	0,183729347	0,041089526
HPGD	-0,12284319	0,233117006	0,000816352	0,992820376
HSD17B10	0,1928242	0,059803447	0,268015736	0,002616506
HSD17B11	0,084820944	0,411266057	0,137856806	0,126795553
HSD17B12	0,407365708	3,80E-05	0,113982691	0,207483505
HSD17B2	0,126030928	0,221119951	-0,03457435	0,703041132
HSD17B4	0,232772653	0,022473593	-0,03270496	0,718402154
HSD17B7	0,152189583	0,183464102	0,186878772	0,037685036
HSD17B8	0,011611503	0,910599432	0,186958595	0,037601926
HSF1	-0,27524893	0,016845943	-0,00885632	0,922231722
HSP90AA1	0,281090613	0,005533693	0,124456334	0,168446675
IGFBP2	-0,14201031	0,167528055	0,012022827	0,894565561
INHBA	-0,33333333	0,346593507	-0,03400668	0,707693414
ITGA2	0,25933261	0,010727401	0,327464988	0,00020525
ITGAM	-0,09526587	0,355858182	-0,13992762	0,12113588
KANK2	-0,22188009	0,029806476	0,025460268	0,778949941
KAT5	-0,3077551	0,029691086	NA	NA
KMT2D	-0,44612046	5,23E-06	0,069064356	0,445946646
KRT19	0,164243082	0,109810376	-0,05666719	0,531885099
LBH	-0,22529817	0,173830825	0,127642523	0,157727006
LCOR	-0,30999435	0,160319651	-0,20626875	0,021537668
MAP1B	-0,24797884	0,014849159	0,171417781	0,056960889
MBD2	0,092506938	0,377813449	0,325224233	0,000228182
MBD3	0,322478955	0,002182752	0,223264304	0,012683531
MDK	-0,12394194	0,228931602	NA	NA
MED1	-0,20578318	0,047827815	-0,17159357	0,056703099

MME	0,169867211	0,11150627	-0,05618577	0,53538179
MMP14	0,001668475	0,987127876	0,102067663	0,259313481
MMP15	0,093911249	0,710899259	0,02618012	0,772867268
MMP19	0,013683634	0,944902978	-0,0493547	0,586196147
MMP2	-0,24442485	0,016394313	0,082435877	0,362706163
MMS19	0,188429192	0,065982404	-0,11472541	0,204518282
NCOA1	0,143967982	0,298985037	-0,06176971	0,495540417
NCOA3	0,006350267	0,972021366	0,09842176	0,27680642
NCOA6	-0,11204482	0,521642688	0,078212383	0,387895976
NCOA7	0,324015009	0,041384094	0,108661015	0,2296333
NCOR2	0,060838782	0,560228833	0,150742098	0,094690815
NR2F2	0,14829083	0,149330644	-0,16134657	0,073416644
NR3C1	-0,16582633	0,341083592	-0,00229001	0,979861767
NRIP1	-0,22322867	0,086445041	0,054158792	0,550228603
OLFM4	0,064812805	0,530413628	-0,06720378	0,458323975
PADI2	-0,13330168	0,195402294	0,009598741	0,915735508
PAK1	-0,01158437	0,910807453	0,264201416	0,003025846
PARP1	0,18640803	0,068992769	0,315612903	0,000356117
PCNA	0,617959848	1,98E-11	0,197186467	0,028153751
PDE3A	-0,11943036	0,25682086	0,070697808	0,435237047
PDGFRA	0,201573521	0,048906871	0,020195324	0,823822915
PELP1	0,223114487	0,028884952	0,075650669	0,403674886
PHB2	0,301180141	0,002865048	0,203084186	0,02368664
PLEKHA1	-0,02252953	0,833064443	0,111184843	0,218929889
POSTN	-0,09363809	0,364174309	0,067820614	0,454199578
PPID	0,13010038	0,206446429	-0,10449161	0,248110989
PPP1R9B	-0,28185024	0,005402169	0,198495673	0,02710462
PRKAA1	0,258247423	0,011072556	0,054055075	0,550993638
PRKCA	-0,2983044	0,003157137	0,03854978	0,670776377
PTN	-0,13544577	0,274458457	NA	NA
PTX3	-0,17289745	0,092072921	0,113630212	0,208901457
RBBP5	0,189894194	0,063867679	0,179125523	0,046526365
RBFOX2	-0,15668667	0,277192426	0,046793135	0,605804172
RERG	0,099244994	0,553303625	NA	NA
RUVBL2	0,464595768	1,86E-06	0,214778914	0,016598593
SAFB	0,143760174	0,162300827	0,047131393	0,603198353
SERPINB9	0,046988606	0,649387851	0,303345397	0,000615327
SERPINF1	-0,07113402	0,491004866	0,310420142	0,000450172
SFRP1	-0,15657612	0,152421744	NA	NA
SGPL1	0,196527401	0,054971255	-0,02490008	0,783692765
SIRT1	-0,02197802	0,943186242	0,119566681	0,18593376
SLC26A6	0,022138837	0,892141608	0,061714186	0,495928709
SLIT3	-0,23776224	0,456800853	-0,06846629	0,449904555
SP1	0,055290288	0,592618672	0,06330008	0,484900856
SRC	0,208505155	0,04148717	-0,1788513	0,046868239
STAT3	0,065179056	0,528087927	0,059210071	0,513607144

STAT5A	-0,204707	0,045427192	0,212003147	0,018088896
STAT5B	-0,16494845	0,108271957	0,213406766	0,017321429
STRN	0,131104178	0,202936482	0,188292683	0,036235537
STRN3	-0,29061313	0,004073882	0,230797797	0,009910305
STXBP1	-0,10042763	0,33027498	0,257680566	0,003860914
SULT1A1	0,071035556	0,501021067	0,033120378	0,714978609
TACC1	-0,30377103	0,002622928	0,311590873	0,000427161
TADA3	0,095073892	0,623722574	-0,01260453	0,889495888
TAF10	-0,06425154	0,578787078	0,29930193	0,000733111
TAF7	0,028713172	0,795426266	0,121088611	0,180354349
TGFB1	-0,27830923	0,136436769	0,028172272	0,756106548
TGFB2	0,738095238	0,036552761	NA	NA
TRIM24	0,234594595	0,044233343	-0,02775155	0,759637088
TRIM25	0,188198589	0,066320391	0,030923682	0,73314452
TRIP4	0,071039067	0,491585166	-0,11803176	0,1916874
TXNIP	0,008362863	0,9398171	0,1420962	0,115420303
UBA5	0,246310364	0,01555808	0,115449434	0,201657143
UBR5	0,221256104	0,030281687	-0,11651298	0,19750673
UFL1	0,022924579	0,824546747	-0,06635405	0,46403936
UFM1	0,141440586	0,169256508	0,202435877	0,024145942
UFSP2	-0,1453337	0,157704658	0,213853659	0,017083072
UGT1A7	-0,32234432	0,101046351	-0,08638881	0,340069796
UGT2B17	0,122965274	0,232649342	-0,19466247	0,030274104
VPS11	-0,2042051	0,04597032	0,238325728	0,007686749
VPS18	-0,09252577	0,369925164	0,230590087	0,009978955
WBP2	-0,36890355	0,000438087	-0,00850525	0,925305446
WIPI1	-0,04453841	0,774057375	-0,08909015	0,325126875
ZNF703	-0,12998643	0,298219454	-0,02557183	0,778006403
ZNF830	-0,00753902	0,958559563	0,00235713	0,979271621
APP	0,249817722	0,014100437	0,116777524	0,196484031
DCN	-0,36994031	0,00020751	-0,0059229	0,947945357
EIF1	0,029571351	0,774873528	0,364173092	3,21E-05
HMGB1	0,173494303	0,090938477	-0,05608183	0,536138258
HNF1A	0,242805559	0,141876872	0,158282619	0,079123244
HSP90AB1	0,519601194	5,80E-08	0,063962234	0,48033556
JUN	-0,10730347	0,322543888	0,140963832	0,118378102
KRT18	0,225990233	0,0268313	-0,00629426	0,944686586
RBP1	-0,12547822	0,290160378	-0,13110936	0,146652041
SERPINE2	-0,00876289	0,93247182	0,068481511	0,449803607
SMAD1	-0,07323657	0,478248647	-0,04824833	0,594629475
TMSB10	-0,3222599	0,001364746	0,060368214	0,505390882
TMSB4X	-0,22641074	0,026541657	0,030357199	0,737853653
METTL7B	NA	NA	0,135194548	0,134367723
PPAN	NA	NA	0,163626522	0,069389794
DEFA1	NA	NA	0,133268569	0,140057559
UGT2B7	NA	NA	-0,10912354	0,227644724

GSTA1	NA	NA	0,062781024	0,488495717
CDKN2B	NA	NA	0,241683824	0,006846249
HPSE2	NA	NA	-0,01133556	0,900560698
MAPK10	NA	NA	0,157902037	0,079856301
MNT	NA	NA	-0,17953855	0,046015364
PLA2R1	NA	NA	-0,09950104	0,271547303
PRMT6	NA	NA	-0,26935811	0,0024848
SPI1	NA	NA	-0,13476723	0,135614651
AKR1B15	NA	NA	0,082196696	0,364105077
ARNT2	NA	NA	-0,14484328	0,108483343
BRCA1	NA	NA	0,090806477	0,315855447
CFLAR	NA	NA	0,107622715	0,234141695
CYP11A1	NA	NA	-0,02896866	0,749437364
CYP3A4	NA	NA	-0,08209021	0,364728955
CYP3A7	NA	NA	-0,0804785	0,374252032
DNMT3A	NA	NA	0,031487669	0,728465966
EPO	NA	NA	-0,08438695	0,351419278
ESRRB	NA	NA	-0,05067567	0,576199479
ESRRG	NA	NA	0,02371609	0,793742925
F7	NA	NA	0,188218046	0,036310861
GHRL	NA	NA	-0,1297292	0,150988475
HOXA10	NA	NA	0,006432606	0,943472769
IHH	NA	NA	-0,10957709	0,225706561
KDM5B	NA	NA	0,19185705	0,032788389
MBD4	NA	NA	0,035071111	0,698978954
NCOA4	NA	NA	0,102027481	0,259502056
NOS3	NA	NA	-0,27623504	0,00189939
NOTCH1	NA	NA	-0,08624463	0,340879351
NOTCH4	NA	NA	-0,0743109	0,412076175
NPR2	NA	NA	-0,0468643	0,60525554
NR3C2	NA	NA	0,088941647	0,325937237
NSD1	NA	NA	-0,08438695	0,351419278
PDE4B	NA	NA	-0,05919682	0,513701555
PGR	NA	NA	0,016373588	0,856764699
PTCH1	NA	NA	0,071791886	0,428146556
RARA	NA	NA	-0,07847491	0,386300174
RGS9	NA	NA	0,036951429	0,683680732
ROBO2	NA	NA	-0,13837315	0,12536568
SLC34A2	NA	NA	-0,14736229	0,102412729
SLIT2	NA	NA	-0,22707882	0,011204446
UGT1A1	NA	NA	-0,08908275	0,325167234
UGT1A3	NA	NA	-0,08908275	0,325167234
UGT2B10	NA	NA	-0,08800006	0,331105324
UGT2B11	NA	NA	-0,07849659	0,386168596
UGT2B15	NA	NA	-0,3198867	0,000292703
UGT2B28	NA	NA	0,036525697	0,687133534

UGT2B4	NA	NA	-0,04634077	0,609296774
ZDHHC21	NA	NA	0,044082737	0,626861178
ZNF366	NA	NA	-0,03400668	0,707693414
ARID5B	NA	NA	0,114930617	0,203704426
GLI1	NA	NA	-0,08438695	0,351419278
INHBB	NA	NA	-0,24044943	0,007145222
LHB	NA	NA	-0,05415879	0,550228603
OTX2	NA	NA	0,137286239	0,128390129
