# Supplementary figures and images for: CK2α Overexpression in Colorectal Cancer: Evidence for Sex- and Age-Linked Differences
Source: Cancers (Basel). 2025 Aug 30;17(17):2857. doi: 10.3390/cancers17172857 (PMC12427210; doi:10.3390/cancers17172857)

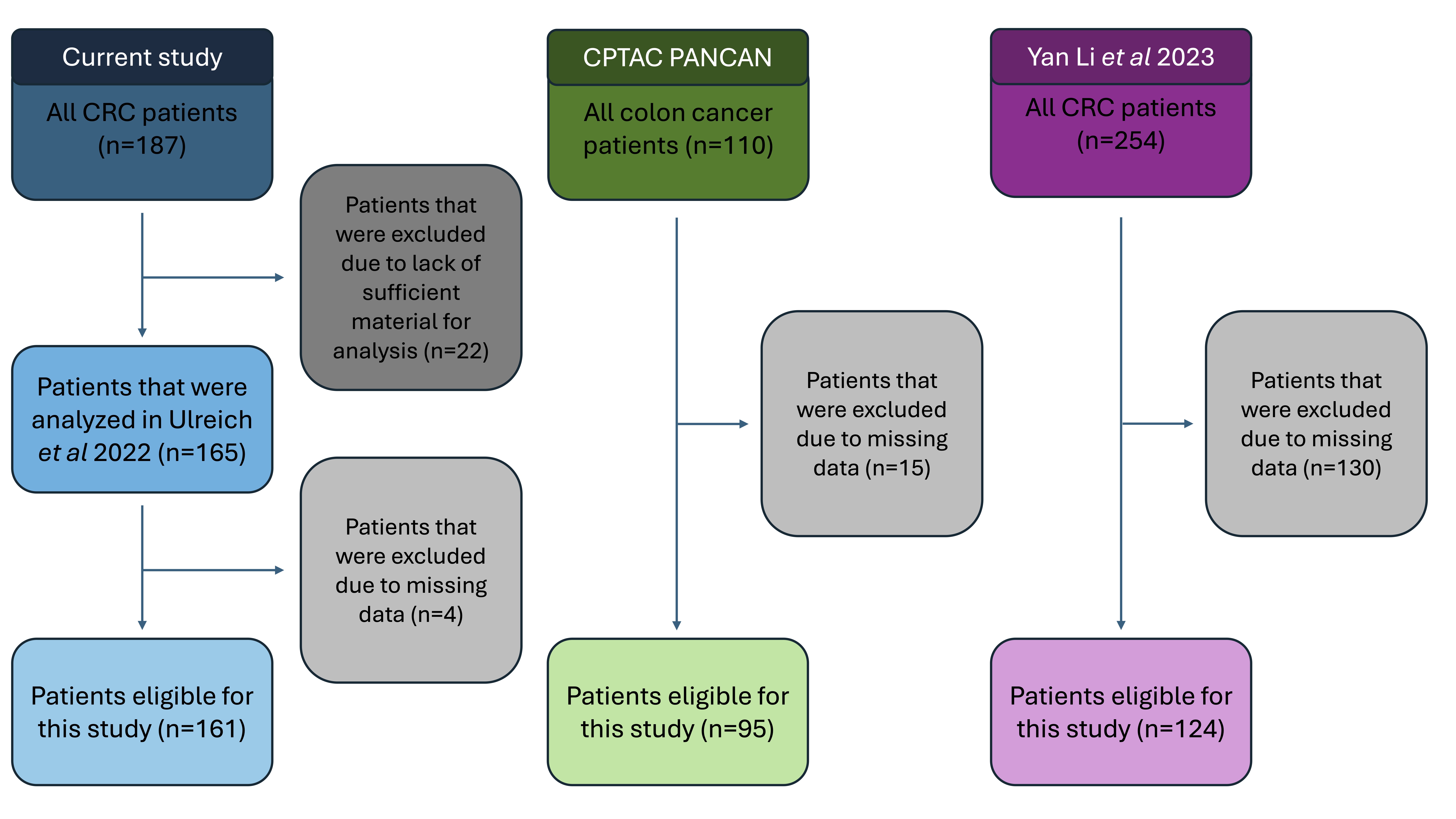

Supplement: Supplementary file 1 [file cancers-17-02857-s001.zip › Supplementary Figure S1.jpg]

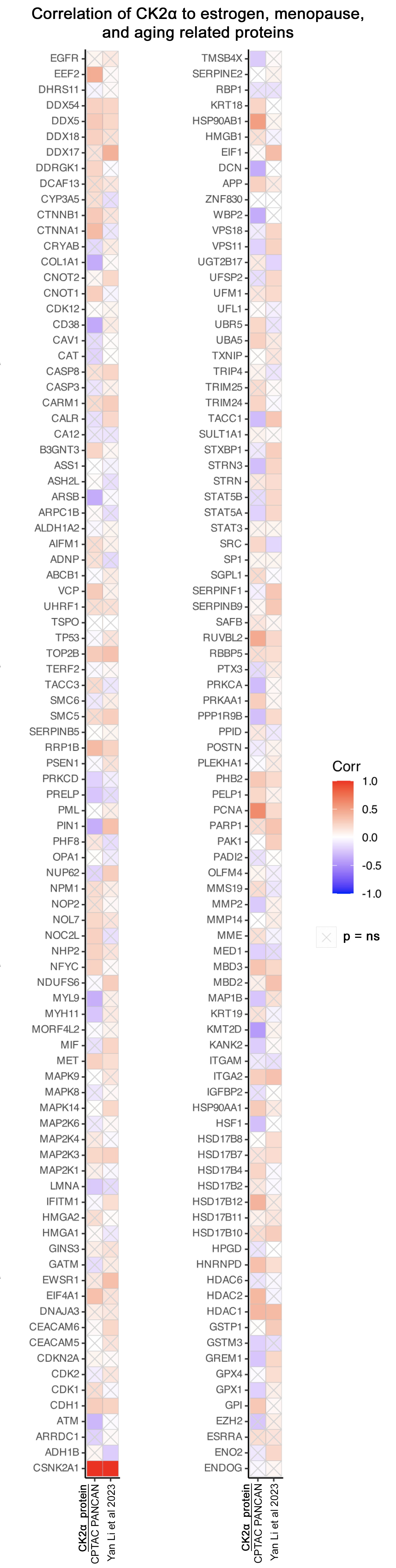

Supplement: Supplementary file 1 [file cancers-17-02857-s001.zip › Supplementary Figure S2.jpg]
